# LSD1-mediated demethylation of OCT4 safeguards pluripotent stem cells by maintaining the transcription of PORE-motif-containing genes

**DOI:** 10.1038/s41598-021-89734-y

**Published:** 2021-05-13

**Authors:** Songsong Dan, Yuelin Song, Xiaotao Duan, Xiao Pan, Cheng Chen, Shiqi She, Tong Su, Jingchao Li, Xinyu Chen, Yanwen Zhou, Wenjie Chen, Xiaobing Zhang, Xiaoyun Pan, Ying-Jie Wang, Bo Kang

**Affiliations:** 1 State Key Laboratory for Diagnosis and Treatment of Infectious Diseases, National Clinical Research Center for Infectious Diseases, Collaborative Innovation Center for Diagnosis and Treatment of Infectious Diseases, The First Affiliated Hospital, School of Medicine, Zhejiang University, Hangzhou, 310003 Zhejiang China; 2 College of Life Sciences, Zhejiang University, 866 Yuhangtang Road, Hangzhou, 310058 Zhejiang China; 3 State Key Laboratory of Toxicology and Medical Countermeasures, Beijing Institute of Pharmacology and Toxicology, Beijing, 100850 China

**Keywords:** Cell biology, Stem cells

## Abstract

Reversible lysine methylation is essential for regulating histones and emerges to critically regulate non-histone proteins as well. Here we show that the master transcription factor OCT4 in pluripotent stem cells (PSCs) was methylated at multiple lysine residues. LSD1 that is highly expressed in PSCs can directly interact with and demethylate OCT4 at lysine 222 (K222) in the flexible linker region. Reduced LSD1 activity led to the methylation of OCT4-K222 that diminished the differentiation potential of PSCs while facilitating proteasome-independent degradation of OCT4 proteins. Furthermore, site-specifically replacing K222 with phenylalanine to mimic the constitutively methylated lysine promoted the ‘locked-in’ mode engagement of the OCT4 PORE-homodimers that tightly bind to and block the transcription of multiple PORE-motif-containing target genes regulating cell fate determination and cell junction organization, and thereby reducing the pluripotency of PSCs. Thus, LSD1-mediated demethylation of OCT4 plays a crucial role in restricting the ‘locked-in’ mode binding of OCT4 PORE-homodimers to the PORE-motif-containing genes and thereby maintaining their transcription to safeguard the pluripotency of PSCs.

## Introduction

Cell fate determination during embryogenesis depends on the properties of self-renewal and pluripotency possessed by pluripotent stem cells (PSCs) including embryonic stem cells (ESCs), embryonal carcinoma cells (ECCs) and induced pluripotent stem cells (iPSCs) that have the potential to differentiate into three embryonic germ layers (endoderm, mesoderm, and ectoderm) and are valuable for basic research, regenerative and translational medicine^1^. Both ESCs derived from the inner cell mass of the mammalian preimplantation blastocyst^2,3^ and their malignant counterpart ECCs derived from teratocarcinomas^4^ are cultured in the Petri dish indefinitely as a unique tool for modeling cell fate determination. In contrast, iPSCs are acquired by enforced expression of certain pluripotency transcription factors, such as OCT4, SOX2, KLF4, and c-MYC (OSKM), in differentiated somatic cells^5,6^. The core stemness circuitry comprising OCT4, SOX2 and NANOG is known to be essential for maintaining the pluripotency and self-renewal PSCs via forming a positive feedback transcriptional regulatory circuit to suppress differentiation^7,8^, and to coordinate with other transcription factors, epigenetic regulators and signaling pathways in cell fate determination^7^. However, little is known about the precise mechanisms underlying the interplay between pluripotency transcription factors and epigenetic regulators in establishing and maintaining the pluripotency of PSCs.

Octamer-binding transcription factor 4 (OCT4, encoded by *POU5F1* gene), a member of the class 5 POU (Pit-Oct-Unc) family of transcription factors, is one of the most important pluripotency transcription factors and pioneer transcription factors participating in ESC maintenance, zygotic gene activation and cellular reprogramming^9,10^. OCT4 proteins specifically bind to the canonical octamer motif (consensus sequence ATGC(A/T)AAT) of target gene enhancer or promoter regions by the cooperation of the POU_S_ (for POU-specific, binding to the sequence ATGC) and POU_H_ (for POU homeodomain, binding to the sequence (A/T)AAT) domains connected with a flexible linker region^10^. This flexible linker of 17 amino acids (N213-A229) allows human OCT4 proteins to form either monomers recognizing the MONO motif (ATGC(A/T)AAT), or OCT4/SOX2 heterodimers binding to the SORE (sox oct recognition element) motif (CATTGTAATGCAAAA), or homodimers in PORE (palindromic octamer recognition element) motif (ATTTGAAATGCAAAT)- or MORE (more palindromic oct factor recognition element) motif (ATGCATATGCAT)-binding configuration, depending on the positioning of POU_S_ and POU_H_ domains relative to each other^9^, and therefore plays a crucial role in regulating the target gene recognition and transcriptional activity of OCT4^9,11,12^. An early study utilizing PORE- and MORE-motif containing luciferase reporters indicated the potential regulation of murine Oct4 homodimer configurations by PKA-mediated phosphorylation of S229 site at the POU_H_ domain^13^, but validation of the conclusion with sufficient number of target genes in the context of PSCs was lacking. A functional study showed that the short α-helix in the linker segment of murine Oct4 could modulate its reprogramming potential by serving as a protein–protein interaction site^12^. Furthermore, the intramolecular interaction between the murine Oct4 linker residues and the POU_H_ RK residues constrained the nonspecific binding of the POU_H_ domain to random DNA sequences to ensure the specific recognition and binding of the OCT4-binding motifs, and it was speculated that post-translational modifications (PTMs) of critical residues in the OCT4 linker may impact on the mode of OCT4-DNA interactions^14^. In the present study, by systematically deciphering and comparing multiple PTM profiles of OCT4 protein in PSCs and other cellular contexts, we identified OCT4-K222 as a conserved crucial residue in the linker region that can be regulated by reversible methylation/demethylation modification. The demethylation of OCT4-K222 by LSD1 played an essential role in safeguarding the pluripotency of PSCs by restricting the ‘locked-in’ mode binding of OCT4 homodimers and allowing for the transcription of a group of PORE-motif-containing genes (PORE genes).

## Results

### K222 site of human OCT4 protein can be methylated in vitro and in vivo

**Figure 1:**
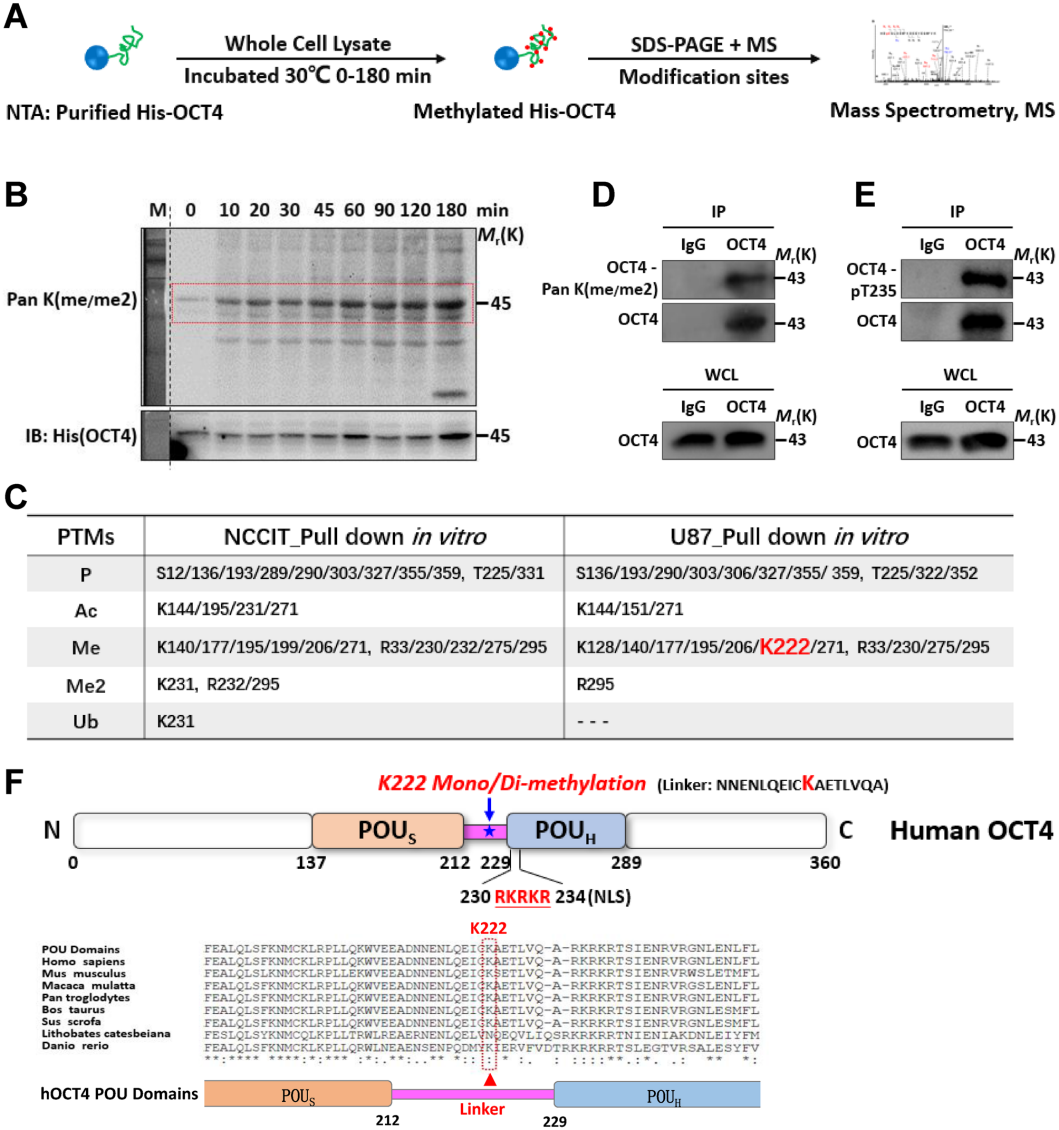
K222 site of human OCT4 protein can be methylated in vitro and in vivo. (**A**) Schematic representation of the cell-free system-based in vitro PTM analysis approach. (**B**) 1 μg of purified E. coli-derived His-OCT4 protein binding to the Ni–NTA beads (20 μl) was incubated with 0.2 mg of NCCIT whole cell lysate in 1 ml PMA buffer at 30℃ for varying periods from 0 to 180 min. Samples were subjected to SDS-PAGE and immunoblotted with the indicated antibodies. (**C**) Summary of the PTM profiles of recombinant His-OCT4 proteins in the cellular contexts of NCCIT and U87 cells. (**D**,**E**) NCCIT whole cell lysates were immunoprecipitated with anti-OCT4, and immunoblotted with anti-pan K(me/me2) (**D**) or anti-OCT4-pT235 (E). Cropped immunoblot images were presented. (**F**) Schematic representation of the OCT4 protein structure, highlighting the methylated K222 site at the linker region that connects the POU_S_ with POU_H_ domains. Multiple alignment of amino acid sequences at the linker region indicated that the K222 is a highly conserved residue across multiple species.

To systematically decipher and compare the PTM profiles of human OCT4 protein, we utilized a cell-free PTM system by incubating bacterially-expressed recombinant OCT4 proteins with the pluripotent NCCIT or differentiated U87 whole cell lysates, followed by purifying the lysate-reacted OCT4 proteins and analyzing their PTMs by mass spectrometry (MS) (Fig. 1A, Figure S1)^15,16^. After incubation with NCCIT cell lysates for 0 to 180 min, the recombinant OCT4 protein samples were subjected to SDS-PAGE and probed by immunoblotting with a pan-mono/di-methylated lysine antibody. Remarkably, recombinant OCT4 proteins were methylated in vitro by the NCCIT lysates in a time-dependent manner and the methylation reaction reached a saturated level at about 60 min of incubation (Fig. 1B), indicating the presence of mono-/di-methylated lysine residues in OCT4 proteins in the context of PSCs. Moreover, endogenous OCT4 proteins immunoprecipitated from NCCIT whole cell lysates can be recognized by an anti-mono-/di-methylated lysine [anti-pan K(me/me2)] antibody (Fig. 1D) and an anti-OCT4-pT235 antibody (Fig. 1E), further confirming the presence of methylated OCT4 proteins in PSCs.

Further MS analyses revealed that multiple lysine and arginine residues in the OCT4 protein can be mono-methylated or di-methylated (Fig. 1C). Notably, mono-methylated K199 and R232, and di-methylated K231 and R232 were preferentially detected in NCCIT-incubated OCT4 proteins, while mono-methylated K128 and K222 were only detected in U87-incubated OCT4 proteins (Figure S1 and S2). In the same set of experiments, other PTMs such as phosphorylation, acetylation and ubiquitination were also detected on multiple residues (Fig. 1C), consistent with the results obtained in previous studies. Comparing with other major PTMs, methylation is probably the least studied PTM for non-histone proteins including OCT4, and therefore, we decided to take a closer look at the methylated residues in the OCT4 protein. Given the crucial role of the linker region in regulating the configuration of OCT4 and its binding mode with various target DNA motifs as mentioned above, it was of particular interest to us that the highly conserved residue K222 is the only positively-charged residue within the entire linker region (Fig. 1F and S4), and differentially methylated in the cellular context of PSCs (NCCIT) vs somatic cells (U87, 293 T) (Fig. 1C, Figure S1, S2, S3 and Table S1).

### LSD1 is a bona fide demethylase for OCT4-K222

**Figure 2:**
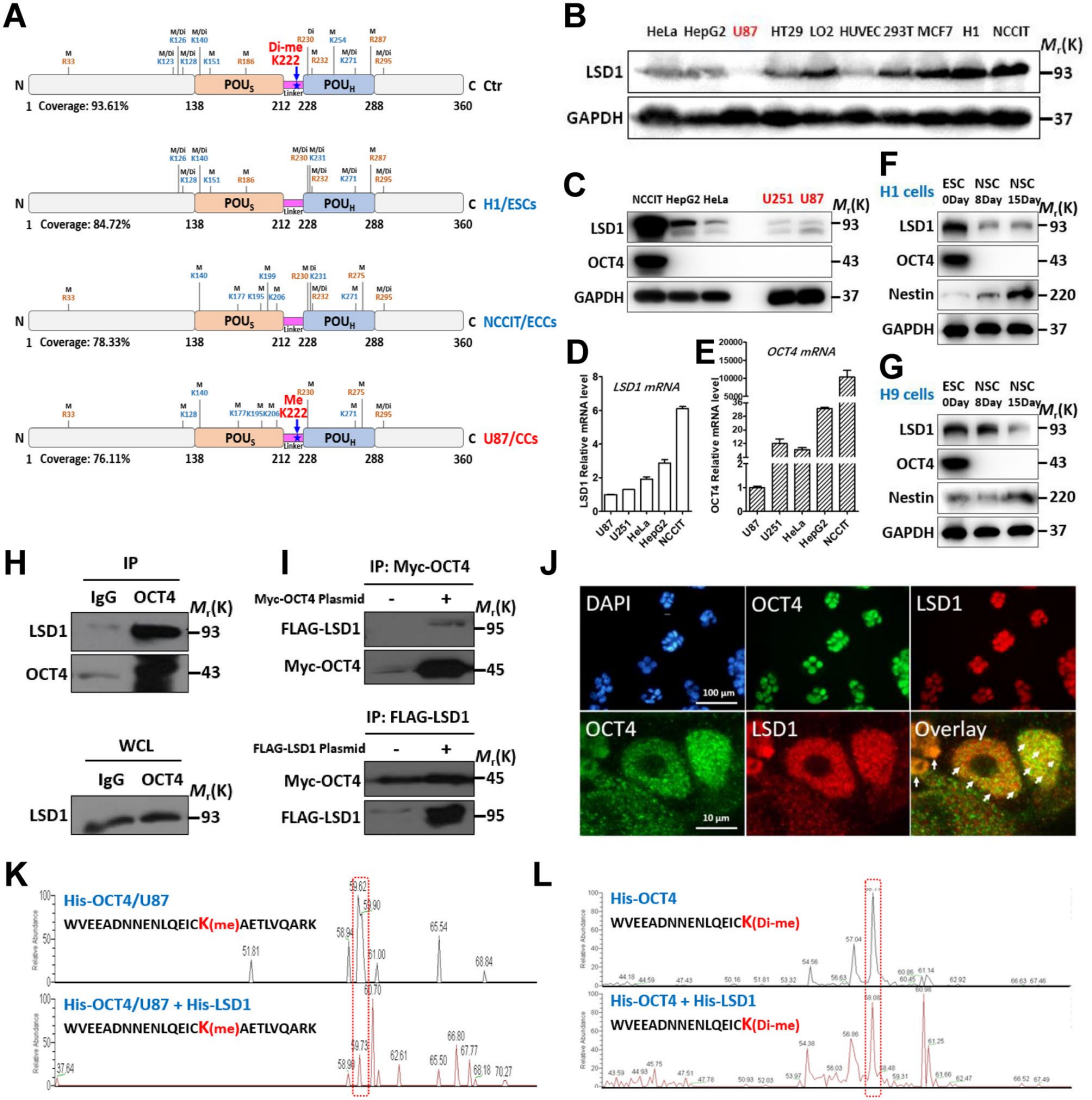
LSD1 is a *bona fide* demethylase for OCT4-K222. (**A**) Comparison of methylated lysine and arginine residues in non-treated recombinant OCT4 proteins with those in recombinant OCT4 proteins incubated with the lysates from different cell types. M, mono-methylation; Di, di-methylation; K, lysine; R, arginine. (**B**) Immunoblotting analysis of LSD1 protein levels in multiple human cell lines that include non-transformed differentiated cells, cancer cells, an ESC and an ECC. (**C**–**E**) Whole cell lysates of multiple cell lines were immunoblotted with anti-LSD1 and anti-OCT4 (**C**), and their LSD1 mRNA levels (**D**) and OCT4 mRNA levels (**E**) were determined by qRT-PCR. (**F**,**G**) H1 (**F**) and H9 (**G**) hESCs were induced to NSCs for 8 and 15 days, and the whole cell lysates were immunoblotted with the indicated antibodies. (**H**,**I**) Endogenous OCT4 proteins in NCCIT cells (**H**) and Myc-OCT4 proteins ectopically-expressed in 293 T cells (**I**) were immunoprecipitated with specific antibodies and immunoblotted with the indicated antibodies. (**J**) Co-localization of endogenous OCT4 and LSD1 proteins in NCCIT cells. White arrows marked the co-localization spots in different cells forming a single colony. (**K**,**L**) Recombinant His-OCT4 proteins were incubated with recombinant His-LSD1 proteins in the presence (**K**) or absence (**L**) of U87 whole cell lysates in PMA buffer for 1 h and subjected for MS analysis. The peak profiles of the peptides spanning the di-methylated and mono-methylated OCT4-K222 were presented. Cropped immunoblot images were presented for (**B**), (**C**), (**F**), (**G**), (**H**) and (**I**).

In a new set of experiment, we systematically compared the methylation profiles of recombinant OCT4 proteins incubated with the whole cell lysates from PSCs (H1 hESC, NCCIT hECC) and somatic cancer cells (U87), with the untreated recombinant OCT4 proteins as control. Consistent with the above results, OCT4-K222 was demethylated in the cellular contexts of both ESCs and ECCs, while OCT4-K222 in U87 cells was mono-methylated and untreated recombinant OCT4 was di-methylated (Fig. 2A, S1 and Table S1). Given OCT4-K222 was demethylated or methylated at very low levels in PSCs, we next searched for the responsible demethylase(s). As LSD1 was known to occupy the promoters of a subset of developmental genes that were co-occupied by OCT4 and NANOG in human ESCs^17^, it is a likely candidate that demethylates OCT4 protein. Consistent with this assumption, LSD1 was found highly expressed in H1 and NCCIT cells but barely detectable in U87 cells (Fig. 2B). Besides U87, another neuronal cell line U251 also had very low levels of LSD1 protein (Fig. 2C) and LSD1 mRNA (Fig. 2D), and there was no correlation between LSD1 and OCT4 expression at mRNA level (Fig. 2D vs E) and protein level (Fig. 2C). When H1 or H9 hESCs were differentiated into neural stem cells (NSCs), there was a significant reduction of the LSD1 protein levels (Figs. 2F,G). Again, there was no correlation between LSD1 and OCT4 protein levels during neural differentiation (Figs. 2F,G). Taken together, we demonstrated that a low level of LSD1 expression in neuronal cells was associated with methylated OCT4-K222, while a high level of LSD1 in PSCs was associated with demethylated OCT4-K222, consistent with the hypothesis that LSD1 is a major demethylase for OCT4-K222. However, even in the presence of LSD1, there are most likely other factors that can affect the probability and efficiency of OCT4-K222 demethylation, and therefore the LSD1 expression level may not always be correlated with the level of methylated OCT4-K222 in various cellular contexts.

Next, we examined the potential physical interaction between LSD1 and OCT4 proteins via Co-IP and co-localization study. The results showed that LSD1 interacted with OCT4 endogenously in NCCIT cells (Fig. 2H) or when ectopically-expressed in 293 T cells (Fig. 2I), and they were partially co-localized in the nucleus of NCCIT cells (Fig. 2J). To determine if LSD1 is able to directly and site-specifically demethylate OCT4-K222, we incubated recombinant OCT4 proteins with recombinant LSD1 proteins in the presence of U87 cell lysate (to provide necessary reaction substrates and co-factors). MS analysis demonstrated that recombinant LSD1 proteins significantly reduced the peak of the OCT4 peptide spanning the mono-methylated K222 (Fig. 2K), suggesting it is a *bona fide* demethylase for OCT4-K222. As expected, when the U87 cell lysate was omitted, recombinant LSD1 proteins were unable to demethylate the di-methylated OCT4-K222 (Fig. 2L).

### K222 methylation promotes proteasome-independent degradation of OCT4

**Figure 3:**
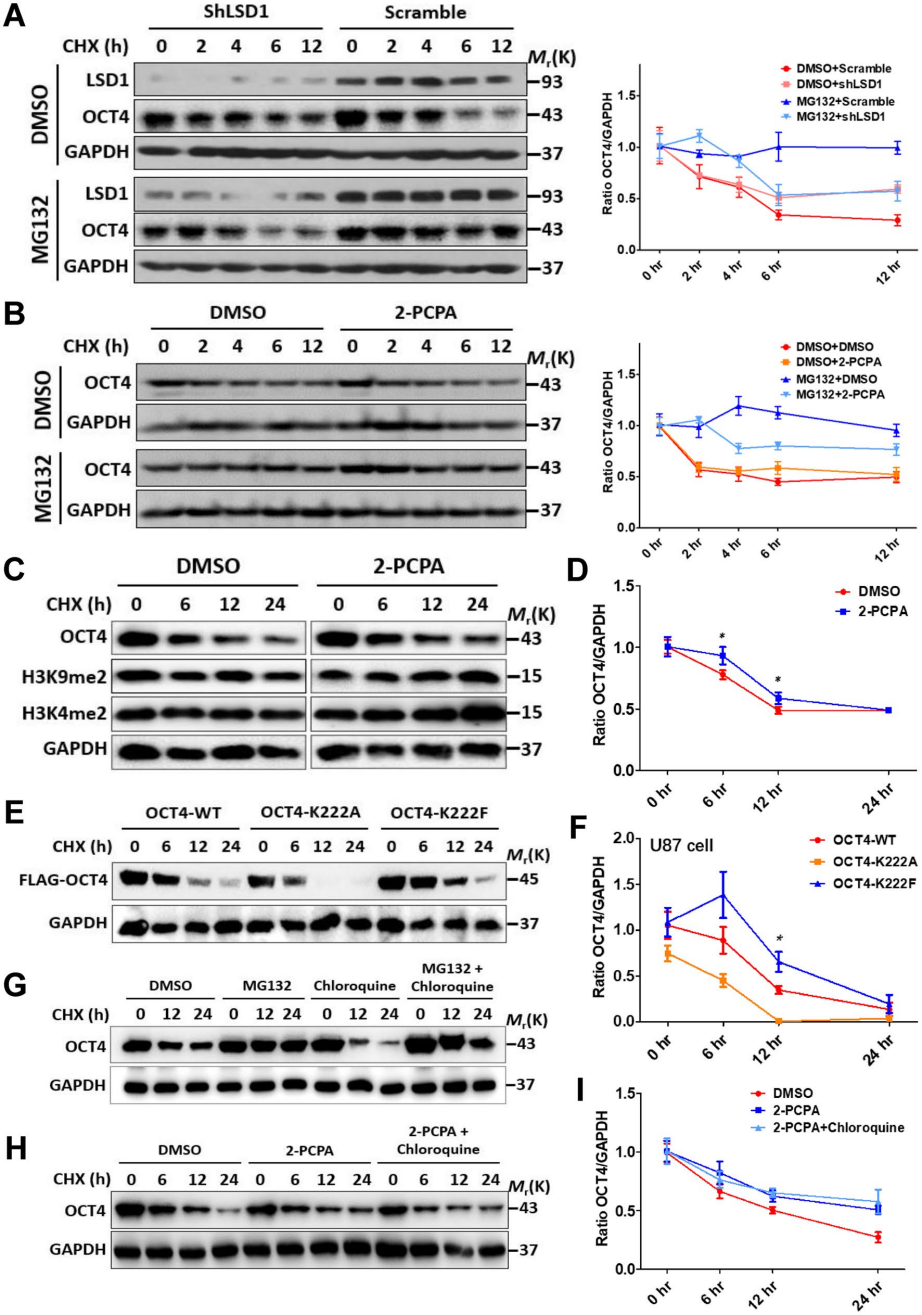
K222 methylation promotes proteasome-independent degradation of OCT4. (**A**) NCCIT cells were infected with LSD1 shRNA lentiviruses. After 96 h, cells were pre-treated with 5 μM MG-132 or vehicle for 1 h, followed by addition of 20 μg/ml CHX and further incubation for 0 to 12 h in the presence of MG-132. Whole cell lysates were immunoblotted with the indicated antibodies. The curves represented quantitation of the OCT4 protein levels. (**B**) NCCIT cells were pre-treated with 100 μM 2-PCPA and 5 μM MG-132 for 1 h and followed by the same steps as described in (**A**). (**C**) NCCIT cells pre-treated with DMSO (left panels) or 2-PCPA (right panels) were exposed to CHX for 0 to 24 h, the whole cell lysates were immunoblotted with the indicated antibodies, and the OCT4 protein levels were quantified in (**D**). (**E**) U87 cells were transfected with the shOCT4-FLAG-OCT4 plasmids (WT and variants). After 72 h, they were treated with 20 μg/ml CHX for 0 to 24 h, the whole cell lysates were immunoblotted with the indicated antibodies, and the OCT4 protein levels were quantified in (**F**). (**G**) NCCIT cells were pre-treated with 5 μM MG-132 or 200 μM chloroquine singly or in combination for 1 h followed by addition of 20 μg/ml CHX and further incubation for 0–24 h. Whole cell lysates were immunoblotted with the indicated antibodies. (H) NCCIT cells were pre-treated with 100 μM 2-PCPA alone or in combination with 200 μM chloroquine for 1 h followed by addition of 20 μg/ml CHX and further incubation for 0 to 24 h. Whole cell lysates were immunoblotted with the indicated antibodies, and the OCT4 protein levels were quantified in (**I**). Results shown in (**D**) and (**F**) were presented as means ± S.D. of triplicate measurements from single experiment representative of 3 independent experiments. Two-tailed unpaired Student’s t tests were used for statistical analyses. *P < 0.05 and **P < 0.01. Cropped immunoblot images were presented for (**A**), (**B**), (**C**), (**E**), (**G**) and (**H**).

Finely-tuned OCT4 protein levels are largely controlled by proteasome-dependent degradation pathway^18,19^. To assess the potential effect of LSD1-mediated demethylation on OCT4 protein turnover, a lentiviral-based shRNA was employed to reduce the endogenous LSD1 protein level in NCCIT cells that were pre-treated with MG-132, a specific and cell-permeable proteasome inhibitor, for 1 h prior to treatment with cycloheximide (CHX) to block synthesis of new proteins. CHX treatment revealed that OCT4 proteins in control cells infected with scramble shRNA had a half-life of approximately 4 h which was increased to 6 h by LSD1 shRNA (Fig. 3A). Strikingly, MG-132 failed to block the degradation of OCT4 proteins in LSD1-silenced cells despite it largely blocked OCT4 degradation in control cells (Fig. 3A), indicating that a lack of LSD1 can promote a proteasome-independent pathway for OCT4 protein degradation. Likewise, when tranylcypromine (2-PCPA) HCl, a specific LSD1 demethylase activity inhibitor, was used to treat NCCIT cells pre-treated with MG-132 and CHX, MG-132 also failed to block the degradation of OCT4 proteins (Fig. 3B). These results indicated that a sufficient LSD1 protein level is required for maintaining the dominant proteasome-dependent OCT4 degradation in PSCs.

To determine the kinetics of OCT4 protein turnover in a longer time frame, NCCIT cells pre-treated with 2-PCPA were exposed to CHX for 0 to 24 h. 2-PCPA treatment slowed down the turnover of both OCT4 and di-methylated H3K9/H3K4 proteins (Fig. 3C,D). To evaluate the effect of site-specific methylation of K222 on the turnover of the OCT4 protein, two site-specific mutants were constructed with the FLAG-OCT4-K222F to mimic the constitutively methylated K222, and the FLAG-OCT4-K222A to represent methylation-deficient K222. These two FLAG-tagged OCT4 mutants along with the FLAG-tagged wild type (WT) OCT4 were incorporated into a shOCT4-containing vector, respectively, and introduced into U87 cells by transfection. CHX treatment showed that, compared with the WT OCT4 proteins, OCT4-K222F proteins were more stabilized while OCT4-K222A proteins were degraded more rapidly (Fig. 3E,F). Like WT OCT4, both OCT4 mutants exhibited a predominant nuclear localization when ectopically-expressed in U87, NCCIT, 293 T (Figure S5), or HeLa (Figure S6) cells.

As for most proteins, lysosome/autophagy-dependent degradation pathway is the major proteasome-independent degradation pathway that can be blocked by chloroquine^15,20,21^, we pre-treated NCCIT cells with chloroquine combined with MG132 or 2-PCPA followed by treatment with CHX for 0 to 24 h. Chloroquine alone speeded up the degradation of OCT4 which can be prevented by MG-132 (Fig. 3G), indicating that there is likely an interplay between the lysosome/autophagy-dependent and the proteasome-dependent degradation of OCT4, and blocking degradation via the former pathway may promote that via the latter one. Importantly, 2-PCPA significantly slowed down the expedited degradation of OCT4 by chloroquine (Fig. 3H,I and S7), further confirming that LSD1-mediated demethylation facilitates the proteasome-dependent degradation of OCT4 protein. Collectively, the enhanced OCT4-K222 methylation derived from either LSD1 silencing or inhibition of LSD1 demethylase activity increased the stability of OCT4 proteins and promoted their degradation via the lysosome/autophagy-dependent pathway.

### OCT4-K222F promotes the ‘locked-in’ mode engagement of OCT4 PORE-homodimers and represses their transcriptional activities in vitro

**Figure 4:**
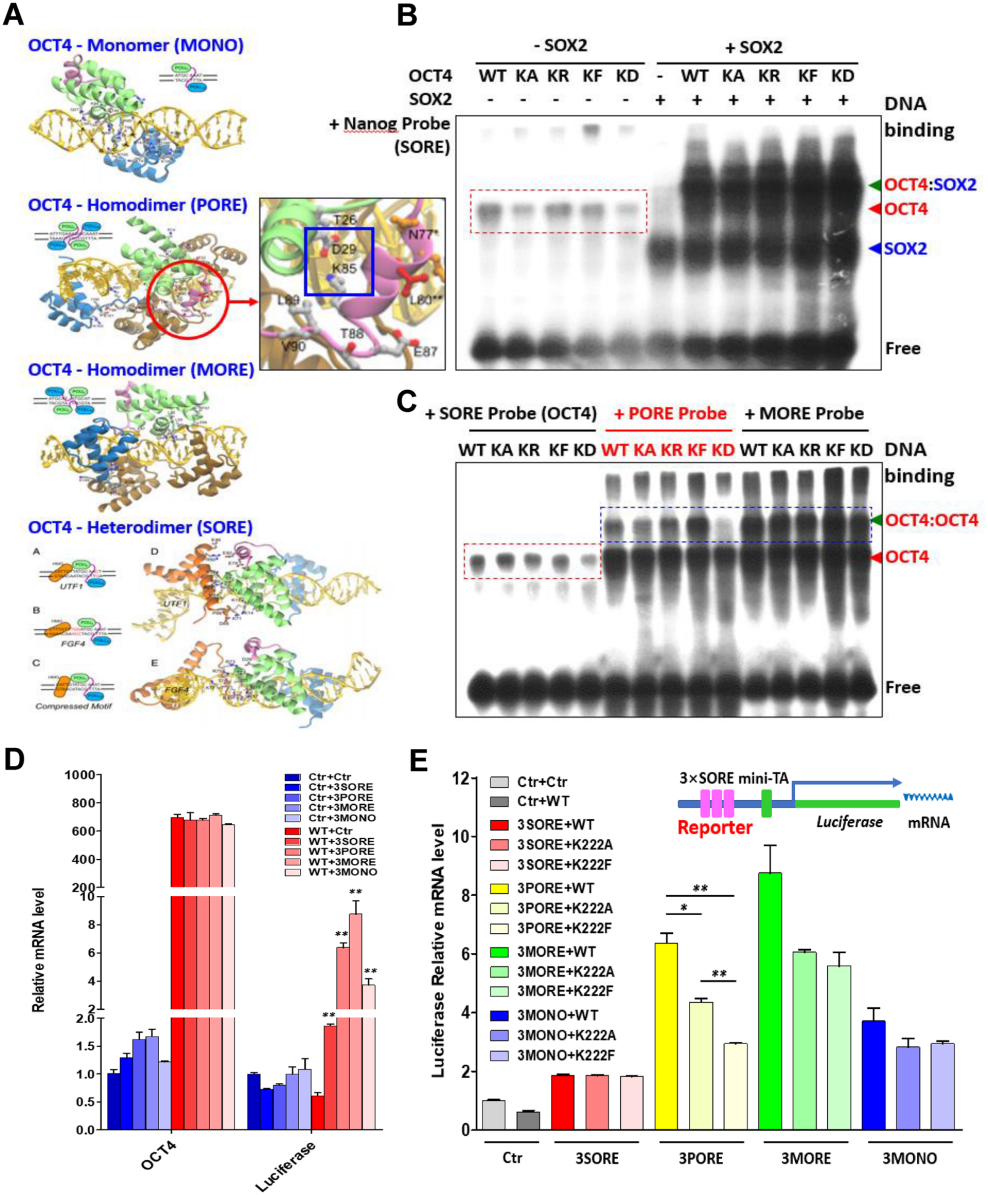
OCT4-K222F promotes the ‘locked-in’ mode engagement of OCT4 PORE-homodimers and represses their transcriptional activities in vitro. (**A**) Models of OCT4 monomer, PORE, MORE homodimer and OCT4/SOX2 heterodimer binding to their specific DNA motifs (pictures adopted from Esch et al., 2013; Jerabek et al., 2014). (**B**) and (**C**) EMSA experiments examining the binding of purified recombinant His-OCT4 (WT and K222 variants) proteins singly or in combination with recombinant SOX2 proteins, to biotinylated SORE, PORE and MORE probes. (**D**) HeLa cells were co-transfected with different OCT4-binding motif-luciferase reporter constructs and the WT OCT4 construct with the empty vector as a control, relative mRNA levels of the expressed OCT4 and luciferase were determined by qRT-PCR 48 h after transfection. (**E**) HeLa cells were co-transfected with different OCT4-binding motif-luciferase reporter constructs and the OCT4 constructs (WT and K222 variants) with the empty vector as a control, the relative luciferase mRNA levels were determined by qRT-PCR 48 h after transfection. Results shown in (**D**) and (**E**) were presented as means ± S.D. of triplicate measurements from single experiment representative of 3 independent experiments. Two-tailed unpaired Student’s t tests were used for statistical analyses. *P < 0.05 and **P < 0.01.

As stated above, OCT4 proteins can form monomers, PORE- and MORE-homodimers, or OCT4/SOX2 heterodimers to regulate the transcription of target genes harboring the MONO, PORE, MORE or SORE motifs, respectively^9,11,12^. A previous structural analysis indicated that positively-charged K222 (numbered as K85 in the structure) can form an intramolecular salt bridge with negatively-charged D166 (numbered D29 in the structure) that may help to stabilize the PORE-mode OCT4 homodimers (Fig. 4A)^12^. To evaluate the impact of K222 methylation on the configuration and transcriptional activity of OCT4, we first conducted the EMSA experiments to determine the in vitro binding among recombinant WT OCT4 protein and its variants (K222A or KA, K222R or KR, K222F or KF, and K222D or KD) with DNA probes containing various OCT4-binding motifs. Among all the tested OCT4 variants, the K222D, when present as monomers, had the lowest binding affinity to NANOG- and OCT4-SORE motifs (Fig. 4B,C and S8). When present as homodimers or heterodimers, K222D proteins minimally bound to the PORE motif (Fig. 4C) but had no obvious impact on binding to the SORE or MORE motifs (Fig. 4B,C). In contrast, K222F homodimers significantly increased the binding to the PORE motif compared to WT and other OCT4 variants (Fig. 4C). These in vitro data strongly suggested that K222 is the crucial residue controlling the configuration of OCT4 homodimers, and constitutively methylated K222 can greatly promote the formation of PORE-mode OCT4 homodimers and strengthen their binding to the PORE genes.

To assess the transcriptional activities of WT OCT4 and the K222 variants, we generated constructs of luciferase reporters whose transcription is driven by three tandem repeats of one of the OCT4 binding motifs (designated as 3SORE, 3PORE, 3MORE and 3MONO, respectively). These OCT4-binding motif-luciferase reporter constructs were co-transfected with OCT4 variant genes into HeLa cells, and the mRNA levels of the transcribed luciferase as well as the OCT4 variants were determined by qRT-PCR. In the first set of experiment where different OCT4-binding motif-luciferase reporter constructs were co-transfected with the WT OCT4 construct only, the level of luciferase transcription driven by the 3MORE was the highest, followed by that of 3PORE, 3MONO, and 3SORE (Fig. 4D), suggesting that in the cellular context of somatic cells, OCT4 MORE- and PORE-homodimers are probably the most favored configurations. Next, OCT4-K222A and OCT4-K222F proteins were compared with the WT OCT4 proteins for their capability of driving the transcription of the same OCT4-binding motifs. There was no difference between WT OCT4 and the two K222 mutants in driving 3MONO- and 3SORE-mediated luciferase transcription, but both K222 mutants moderately reduced the 3MORE-luciferase transcription to the same extent (Fig. 4E). Most interestingly, compared with the WT OCT4, OCT4-K222F significantly reduced the 3PORE-mediated luciferase transcription by greater than 50% while the inhibitory effect of OCT4-K222A was less prominent (Fig. 4E). This observation indicated that constitutively-methylated K222 residue may stabilize the configuration of OCT4 PORE homodimers and lead to their prolonged binding to the PORE genes in a ‘locked-in’ mode and thereby suppressing the transcription of those genes.

### OCT4-K222 methylation represses the transcription of PORE-motif-containing target genes in vivo and the differentiation capacity of PSCs

**Figure 5:**
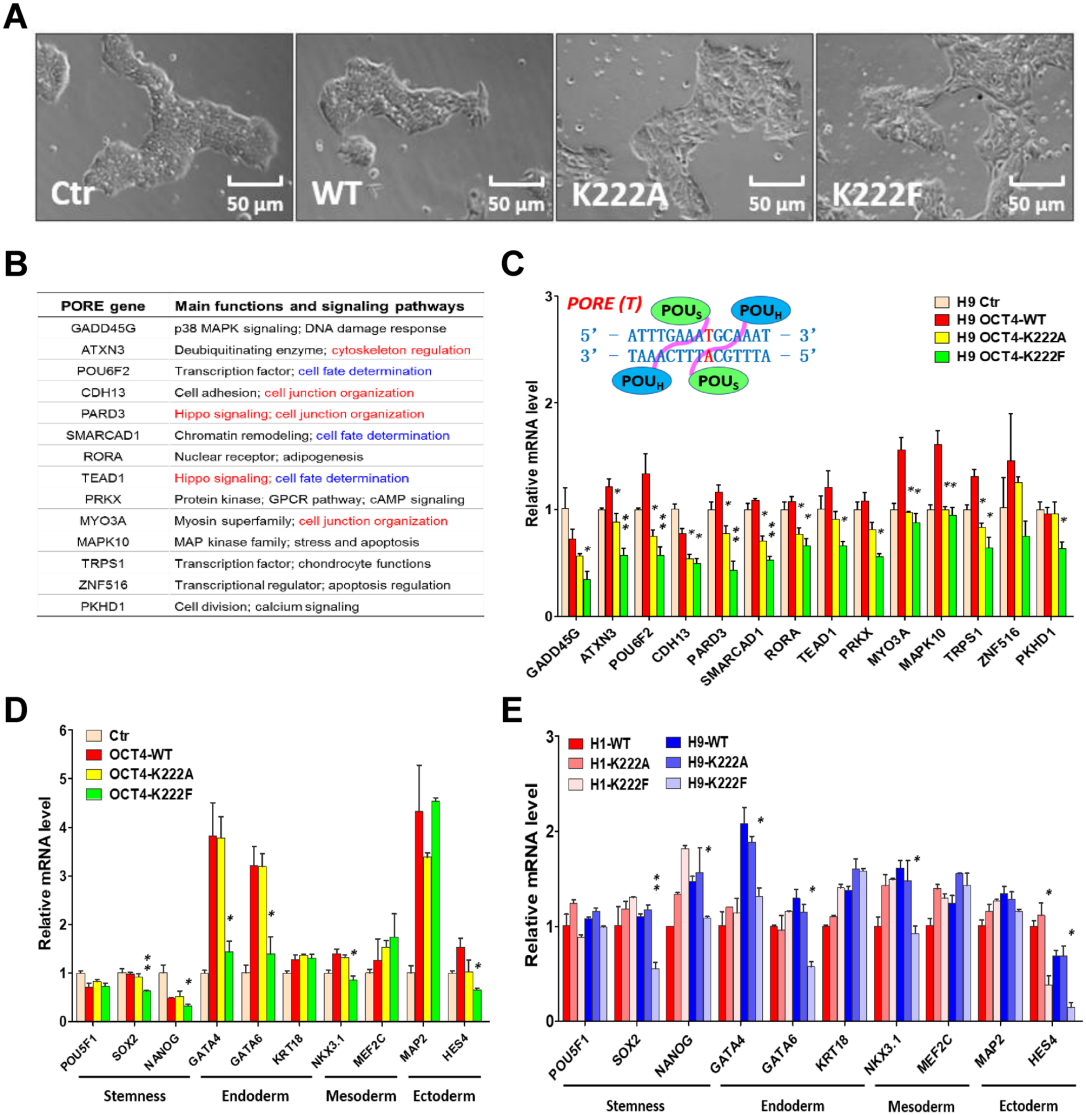
OCT4-K222 methylation represses the transcription of PORE-motif-containing target genes in vivo and the differentiation capacity of PSCs. (**A**) The morphology of H9 cells 72 h after transfection with shOCT4-FLAG-OCT4 (WT and variants) constructs. (**B**) A brief summary of PORE genes’ main functions. (**C**) H9 cells were transfected with shOCT4-FLAG-OCT4 (WT and variants) constructs with the empty vector as a control. 72 h later, cells were harvested and the relative mRNA levels of the PORE genes listed in (**B**) were determined by qRT-PCR. (D,E) The relative mRNA levels of the stemness genes and lineage-specific marker genes in H9 (**D**) and H1 (**E**) cells subjected to the same transfection procedures as in (**C**) were determined by qRT-PCR. Results shown in (**C**), (**D**) and (**E**) were presented as means ± S.D. of triplicate measurements from single experiment. Two-tailed unpaired Student’s t tests were used for statistical analyses. *P < 0.05 and **P < 0.01.

To further explore the physiological functions of OCT4-K222 methylation in the context of PSCs, we transfected H9 human ESC cells with the shOCT4-FLAG-OCT4 constructs expressing WT OCT4, OCT4-K222A or OCT4-K222F, respectively, while knocking down endogenous OCT4 simultaneously. Compared with H9 cells transfected with the control vector and WT OCT4 which maintained compact colonies, K222A-, and particularly K222F-transfected H9 cells became flattened and the colonies were also loosened (Fig. 5A). Given K222 methylation promoted the formation and engagement of the OCT4 PORE-homodimers, we examined its impact on the transcription of PORE genes in PSCs. By nucleotide BLAST searching, we came up with a list of PORE genes that contain one or more PORE motif sequences (ATTTGAAATGCAAAT)^9^ (Table S2). The main functions of the PORE genes center on cell fate determination, cell junction/cytoskeleton organization and regulation of signaling pathways such as the Hippo, MAP, and calcium signaling pathways (Fig. 5B, Table S4). Remarkably, OCT4-K222F dramatically reduced the transcription of all the PORE genes in ESCs and OCT4-K222A also exhibited some suppressive effects (Fig. 5C and S9), consistent with the results obtained in HeLa cells co-transfected with OCT4-K222 mutants and different OCT4-binding motif-luciferase reporter constructs (Fig. 4E). As a control, we examined the transcription of multiple genes harboring the PORE-like-motif (ATTTGAAAGGCAAAT) that only differs the PORE motif (ATTTGAAATGCAAAT) by one single nucleotide (Table S2 and S6). Strikingly, the transcription of most PORE-like-motif-containing genes was only moderately inhibited by two OCT4-K222 mutants to the same extent (Figure S10), suggesting that the recognition of the PORE motif by the OCT4 PORE-homodimers is highly specific, and the suppression of the PORE genes by the OCT4-K222F mutant is likely to be specific and physiologically relevant.

Next, we examined the transcription levels of some key stemness genes and three germ layer marker genes in the above H9 cells (female, XX) and H1 cells (male, XY) subjected to similar treatments. The expression of SOX2, NANOG, GATA4, GATA6, NKX3.1 and HES4 was reduced by OCT4-K222F and, to a lesser extent, by OCT4-K222A in H9 cells (Fig. 5D,E), consistent with the partially-differentiated morphology of the cells and colonies (Fig. 5A). In contrast, under the same conditions, only HES4 transcription was repressed in H1 cells (Fig. 5E). After searching for potential PORE-motif-like sequences in all the above stemness and germ layer marker genes, we could find no single sequence in any genes that completely matches that of the PORE motif (Table S3), indicating that the reduced expression of some genes seen in Fig. 5D,E is most likely a secondary event caused indirectly by OCT4-K222F.

Retinoic acid (RA) is a vitamin A metabolite that is essential for early embryonic development and promotes stem cell neural lineage specification at relatively high concentrations^22^. It is widely used for inducing the differentiation of cultured PSCs into embryoid bodies and neural ectodermal cells. To track transcriptional changes of the PORE genes during RA-induced differentiation of ECCs, NCCIT cells were treated with RA for 0, 3, and 9 days, respectively. Most PORE genes showed time-dependent suppression in transcription (Figure S11), consistent with the assumption that transcription of the PORE genes is positively associated with the self-renewal and pluripotency of PSCs.

We further investigated the effects of two LSD1 inhibitors, 2PCPA and OG-L002 which presumably increase the OCT4-K222 methylation, on RA-induced differentiation of NCCIT cells. NCCIT cells were pre-treated with LSD1 inhibitors and RA for 1 h followed by adding in CHX and a further incubation for 12 h (Figure S12A). Cells treated with the two LSD1 inhibitors showed similar patterns of OCT4 protein degradation and H3K9 methylation (Figure S12B). CHX treatment per se appeared to elevate the expression of mesendoderm marker genes (Figure S12C). Interestingly, the overall transcription patterns of the lineage-specific markers in NCCIT cells treated with the more potent LSD1 inhibitor OG-L002 were similar to those in H9 cells transfected with the OCT4-K222F mutant, supporting the assumption that OCT4-K222 methylation is likely a common target for these two experimental settings.

## Discussion

Accumulating evidence indicates that the protein stability, transcriptional activity, subcellular localization, protein–protein interaction of OCT4 are comprehensively and delicately regulated by a variety of PTMs including phosphorylation^13,15,21,23^, O-GlcNAcylation^24^, sumoylation^25^, ubiquitination^19,26,27^, and acetylation^28^. Although methylation of non-histone proteins at lysine residues emerges to influence their stability and functionality, regulate their interaction with DNA and partner proteins, and prevent other PTMs at the same lysine residues^29^, systematic identification and in-depth functional characterization of methylated residues in OCT4 proteins is still lacking. In this study, we identified multiple methylated lysine and arginine residues in OCT4 proteins in the cellular contexts of PSCs and somatic cancer cells, and demonstrated that LSD1 can maintain the un-methylated status of the crucial OCT4-K222 residue to prevent the ‘locked-in’ mode engagement of the OCT4 PORE-homodimers and thereby allowing for the transcription of the PORE genes.

LSD1 was the first identified histone lysine-specific demethylase that demethylates H3K4 or H3K9 in a reaction utilizing flavin adenosine dinucleotide (FAD) as a cofactor^30^. It represses a variety of genes via demethylation of H3K4me1/me2^30^. In human ESCs, high levels of LSD1 are required to suppress H3K4 methylation at the regulatory regions of differentiation genes and thereby maintaining their undifferentiated state, and downregulation of LSD1 during differentiation can favor the resolution of the bivalent domains towards H3K4 methylation and gene activation^17^. Interestingly, when forming a complex with androgen receptor, LSD1 changes its substrates to H3K9me2 and can regulate gene activation through demethylation of H3K9me1/me2^31^.

Besides histone family proteins, LSD1 is able to demethylate lysine residues at a growing number of non-histone substrates^32^, such as p53^33^, DNMT1^34^, STAT3^35^, E2F1^36^, and HIF1α^37^. Given the finding that there is a significant overlap of genome-wide distribution of OCT4 binding sites with the LSD1 binding sites^17^, and OCT4 is associated with different subunits of the CoREST and NuRD complexes^38–40^, OCT4 may recruit LSD1 to target genes via direct or indirect interactions. In this study, we demonstrated direct interaction between LSD1 and OCT4 in PSCs, and the methylated OCT4-K222 site can be demethylated by LSD1 both in vitro and in vivo. Recently, it was shown that murine Oct4 can interact with Lsd1 to inhibit its catalytic activity in F9 ECCs, leading to retention of H3K4me1 and a “primed” chromatin state at pluripotency gene enhancers^41^. It remains to be seen if such inhibitory mechanism is also present in ESCs, and if this may represent a feedback regulation between OCT4 and LSD1.

Among multiple methyl-lysine sites identified in OCT4, K222 is of particular interest for the following reasons: firstly, it is situated at the highly flexible linker region (N213-A229) connecting the POU_S_ domain with POU_H_ domain that functions as a protein–protein interaction interface and plays a critical role during reprogramming by recruiting epigenetic modifiers to OCT4 target genes^12,42^. Secondly, it is highly conserved across many species despite its neighboring residues are not conserved. Thirdly, it is the only positively charged residue in the whole linker region. Based on the solved and modeled crystal structures^9,12^, the positively charged K222 (numbered as K85 in the cited reference) may be important for forming the intramolecular salt-bridge with the negatively-charged D166 (D29 in the cited reference) to facilitate forming the OCT4 PORE homodimers. Fourthly, although in this study methylation appears to be the only PTM for K222, the information in the PhosphoSitePlus database provided by Cell Signaling Technology, Inc. indicated that murine Oct4-K215 (corresponding to human OCT4-K222) can be ubiquitinated. Therefore, different PTMs may crosstalk at the OCT4-K222 site. For instance, methylation of K222 would be expected to block its ubiquitination, which may well explain our observation that K222 methylation induced by LSD1 ablation promotes the proteasome-independent degradation of OCT4, supporting the notion that methylation increases the stability of proteins by competing with ubiquitination^43^. As methylation of a lysine residue does not modify the side chain's positive charge^43^, the above-mentioned intramolecular salt-bridge between K222 and D166 is unlikely to be disrupted by K222 methylation. However, since the phenylalanine to mimic methylated lysine does not carry any charge, K222F mutant is expected to lose the salt-bridge interaction with D166. Nevertheless, our biochemical data indicated that K222F stabilized the OCT4 PORE-homodimers and strengthened their ‘locked-in’ mode engagement onto the PORE genes and thereby continuously suppressed their transcription. Further structural-based studies are warranted to better understand the detailed molecular mechanisms for such a ‘locked-in’ mode engagement. Paradoxically, K222A mutant that has reduced protein stability and diminished binding with the PORE genes also suppressed their transcription to some extent. These seemingly contradicting results may be reconciled by hypothesizing that a dynamic ‘on-and-off’ mode binding of the OCT4 PORE-homodimers to the PORE genes is required for their normal transcription.

Since its first characterization over two decades ago^11^, the canonical PORE motif sequence (ATTTGAAATGCAAAT) has been analyzed mainly for its interaction with the OCT4 homodimers structurally and biochemically^12,13,44,45^. Surprisingly, few studies have been reported to systematically investigate the physiological functions of the PORE genes and their regulation by OCT4 in PSCs. We show here that the PORE genes are mainly involved in cell fate determination, cytoskeleton/cell junction organization, and a variety of related signaling pathways that are crucial for PSC self-renewal. Importantly, we reveal that the ‘locked-in’ mode engagement of the OCT4 PORE-homodimers inhibits rather than activates the transcription of most PORE genes in PSCs, and LSD1 can prevent such engagement by maintaining the un-methylated status of the crucial OCT4-K222 residue (Figure S13). Taken together, we provide evidence in this study that LSD1-mediated demethylation of OCT4-K222 plays a crucial role in safeguarding the pluripotency of PSCs by restricting the ‘locked-in’ mode engagement of the OCT4 PORE-homodimers to maintain the transcription of the PORE genes. These results reveal a novel interplay between an epigenetic regulator and a pluripotency factor, and exemplify how a site-specific PTM of OCT4 critically regulates the pluripotency of PSCs.

## Methods

### Cell culture, transfection and treatment

The sources and culture conditions for 293 T, HeLa, HepG2, U87, HT29, LO2, HUVEC, MCF7, H1, H9 and NCCIT cells were described in detail previously^16,46,47^. All cells were maintained in a 37 °C incubator with 5% CO2, and free of mycoplasma contamination based on routine tests every 3 months. H1 and H9 cells were cultured in mTeSR1 medium from STEMCELL (catalog no. 85850). 293 T, HeLa and U87 cells were transfected with GenEscort II Transfection Reagent reagents (catalog no. WIS 2100) as described from WISGEN. H1 and H9 cells were transfected with Lipofectamine 3000 Transfection Reagent (catalog no. L3000015) as described in the instructions provide by Thermo-Fisher. In some experiments, cells were treated with 20 μg/ml CHX, 5 μM MG-132, 200 μM Chloroquine, 10 μM Retinoic acid (RA), 100 μM Tranylcypromine (2-PCPA) HCl and 50 μM OG-L002 (DMSO as the vehicle) either individually or in combination.

### Antibodies and reagents

The anti-Oct-3/4 (C-10) mouse antibody (catalog no.sc-5279), anti-Oct-3/4 (H-134) rabbit antibody (catalog no. sc-9081), anti-LSD1 (B-9) antibody (catalog no. sc-271720) and anti- Histone H3 (C-16) antibody (catalog no. sc-8654) were purchased from Santa Cruz Biotechnology. Anti-DYKDDDDK-tag mouse antibody (catalog no. A00187) and anti-DYKDDDDK-tag rabbit antibody (catalog no. A01870), anti-His antibody (catalog no. A00186), anti-GAPDH (HRP) antibody (catalog no. A00192), goat anti-mouse IgG antibody (H&L) [HRP] (catalog no. A00160) and goat anti-rabbit IgG antibody (H&L) [HRP] (catalog no. A00098) were from GenScript. Anti-Myc-tag mouse antibody (catalog no. AM926), anti-Myc-tag rabbit antibody (catalog no. AM933) and di-methyl-histone H3 (Lys9) antibody (catalog no. AF2314) were from Beyotime. Anti-pan-methylated lysine(di-/mono-methyl) antibody (catalog no. ab23366) was from Abcam. Anti-mouse IgG DyLight 488 (catalog no. 715–485-150) and anti-mouse IgG DyLight 594 (catalog no. 715–515-150) were from Jackson ImmunoResearch. Cycloheximide (catalog no. 239764) and MG-132 (catalog no. 474790) were purchased from Calbiochem. Anti-FLAG M2 Magnetic Beads (catalog no. M8823-1ML) was from Sigma-Aldrich. Pierce protein A/G agarose (catalog no. 20421) was from Thermo-Fisher. Ni–NTA agarose resin was from QIAGEN (catalog no. 30310). Chloroquine (catalog no. C6569) was from Sangon Biotech. Retinoic acid (RA) (catalog no. 302–79-4) was from Sigma-Aldrich. Tranylcypromine (2-PCPA) HCl (catalog no. S4246) and OG-L002 (catalog no. S7237) were from Selleck.

### Plasmids and viral infection

The prokaryotic human His-OCT4 and His-SOX2 were generated by PCR and subcloned into the pET28a vector via EcoRI and SacI sites, and His-LSD1 (852 AA) was generated via NheI and XhoI sites. Lentiviral vector was constructed targeting the 3’-UTR of human POU5F1 mRNA to knock down the endogenous OCT4 while expressing the FLAG-tagged exogenous OCT4 WT (referred to as “shOCT4 + FLAG-POU5F1-WT”) was described previously^15^, which was applied to infect and transfect the cells for 48–96 h as described in detail previously^15^. The OCT4 variants (K222A, K222R, K222F and K222D) were generated from OCT4 WT (His-OCT4 or shOCT4 + POU5F1) plasmids by site-directed mutagenesis and applied to the experiments. The FLAG-OCT4-6His portion was subcloned into the EcoRI and XbaI sites of the pLKO.1-TRC-shOCT4-FLAG-OCT4 plasmid^15^.

### Immunoprecipitation and immunoblotting

Immunoprecipitation and immunoblotting were conducted as described previously^15,16^. 1 mg whole cell lysates (293 T and NCCIT) were incubated with 50 μl anti-FLAG M2 magnetic beads or 50 μl Pierce protein A/G agarose over 8 h at 4℃ respectively. 1 mg of whole cell lysates (U87, NCCIT and H1) was incubated with 100 μl of Ni–NTA agarose resin over 2 h at 4℃ respectively. Immunoblotting and Coomassie Brilliant Blue staining were performed after SDS-PAGE. In most cases, cropped immunoblot images were presented in the main figures, with their uncropped or partly-cropped images being shown in the Supplementary Information.

### Immunofluorescence staining and co-localization study

Immunofluorescence staining was conducted as described previously^15^. Briefly, anti-OCT4 and anti-LSD1 antibodies were applied for immunofluorescence staining and their co-localization in NCCIT cells was determined by confocal microscopy (Carl Zeiss LSM-510).

### Electrophoretic mobility shift assay, EMSA

EMSAs were carried out using a LightShift chemiluminescent EMSA kit (Thermo-Fisher, catalog no. 20148) as instructed by the manufacturer. Briefly, a 20 μl reaction mixture containing 1.5 μg purified recombinant His-OCT4 (WT and variants), 0.5 μg purified recombinant His-SOX2, 50 nM 5’-biotin end-labeled dsDNA probes (NANOG-SORE, OCT4-SORE, PORE, MORE) and 1 μl Poly (dl·dC) in 1 × Loading buffer was incubated for 30 min at 25℃. The sequences of the probes were as follows:

NANOG-SORE probe,

Sense:5’-biotin-GTCTGGGTTACTCTGCAGCTACTTTTGCATTACAATGGCCTTGGTGAGACTGGTAGACG-3’.

Anti-sense:5’-CGTCTACCAGTCTCACCAAGGCCATTGTAATGCAAAAGTAGCTGCAGAGTAACCCAGAC-3’.

OCT4 SORE probe,

Sense:5’-biotin-CCGTCTTCTTGGCAGACAGCAGAGAGATGCATGACAAAGGTGCCGTGATGGTTCTGTCC-3’.

Anti-sense:5’-GGACAGAACCATCACGGCACCTTTGTCATGCATCTCTCTGCTGTCTGCCAAGAAGACGG-3’.

OCT4 PORE probe,

Sense:5’-biotin-TATACTAAGCAATTCTTCATTGATTTGAAATGCAAATTTGACTGGGCACCCTGTATCTT-3’.

Anti-sense:5’-AAGATACAGGGTGCCCAGTCAAATTTGCATTTCAAATCAATGAAGAATTGCTTAGTATA-3’.

OCT4 MORE probe,

Sense:5’-biotin-TGTGAAATACCCTGCCTCATGCATATGCAAATAACCTGAGGTCTTCTGAGATAAATATA-3’.

Anti-sense:5’-TATATTTATCTCAGAAGACCTCAGGTTATTTGCATATGCATGAGGCAGGGTATTTCACA-3’.

### Quantitative real-time PCR, qRT-PCR

Quantitative real time PCR analysis and primers used for lineage marker quantitation were as described previously^15^. Firstly, total RNA was extracted by RNAiso Plus (TaKaRa, catalog no. 9109). Secondly, cDNA was synthesized using PrimeScript RT reagent kit with gDNA eraser (TaKaRa, catalog no. RR047A) according to the manufacturer’s instructions. Thirdly, qRT-PCR was performed using the iTaq Universal SYBR Green Supermix (Bio-Rad, catalog no. 1721–5124) in an ABI 7500 Real-Time PCR instrument. All the PCR amplifications were performed in triplicates and repeated in three independent experiments. The relative quantities of mRNAs were normalized by the mRNA levels of the housekeeping gene PBGD (HMBS). The sequences of all RT-PCR primers were listed in Table S5.

### Luciferase reporter-based OCT4 transactivity assay

The pGL6-TA vector (Beyotime, catalog no. D2105) was inserted with three tandem repeats of one of the OCT4 binding motifs (3SORE, 3PORE, 3MORE and 3MONO) via XhoI and HindIII sites. The resulting constructs together with WT OCT4 or OCT4 variant plasmids were co-transfected into HeLa cells, and the luciferase mRNA expression levels driven by the OCT4 binding motifs were determined by qRT-PCR and normalized by the GAPDH mRNA levels. The sequences of the three tandem repeats of OCT4 binding motifs were as follows:

3SORE, 5’-TTTTGCATTACAATGTTTTGCATTACAATGTTTTGCATTACAATG

3PORE, 5’-ATTTGAAATGCAAATATTTGAAATGCAAATATTTGAAATGCAAAT

3MORE, 5’-ATGCATATGCAAATATGCATATGCAAATATGCATATGCAAAT

3MONO, 5’-ATTTGCATATTTGCATATTTGCAT

### Recombinant protein expression and purification

The His-tagged human OCT4 (WT and variants) and SOX2 constructs were transformed into Rosseta E. coli strains and the purification procedures were as described previously^15^. After sonication, the 15 ml E. coli lysate was centrifuged at 12,000 g for 10 min at 4℃ and the supernatants were mixed with 1 ml of 50% (w/v) slurry of Ni–NTA Agarose resin/beads incubated at 4℃ on a rotary shaker for 2 h. The mixture was then centrifuged at 800 g for 2 min and the supernatant was discarded. The saving beads were washed with lysis buffer containing 50 mM imidazole and the proteins binding on Ni–NTA beads were used for subsequent in vitro PTMs assay (prokaryotic proteins eluted with the same buffer containing 200 mM imidazole for EMSA experiments).

### In vitro PTM of recombinant OCT4 proteins

The procedures were largely similar as described previously^16^. An aliquot (5 μg) of purified recombinant His-OCT4 protein binding to the Ni–NTA beads (100 μl) was incubated with 1 mg of the whole lysate derived from NCCIT, H1 or U87 cells at 30℃ for 1 h in 1 ml PMA buffer (50 mM Tris–HCl, pH 7.4, 50 mM KCl, 5 mM MgCl2, 0.5% NP-40, 25 mM imidazole, 5 mM ATP, 100 μM S-adenosyl methionine, 100 μM acetyl-CoA, 1% EDTA-free protease inhibitor cocktail and phosphatase inhibitor cocktail) respectively. 2 μg purified recombinant His-LSD1 protein were added in the demethylation array. The His-OCT4 conjugated beads were sedimented by centrifugation at 800 g for 2 min, washed three times with ice-cold PMA washing buffer (50 mM Tris–HCl, pH 7.4, 50 mM KCl, 5 mM MgCl2, 0.5% NP-40, 50 mM imidazole), eluted and denatured in SDS–PAGE sample loading buffer by heating at 100 ℃ for 5 min. Pooled samples (10–20 μg) were loaded and separated by SDS–PAGE and stained with Coomassie brilliant blue R250. After de-staining, the OCT4 bands (with a molecular weight of 45 kDa) were excised and analyzed by mass spectrometry.

### Mass spectrometric identification of PTMs in OCT4 proteins

OCT4 samples were subjected to overnight digestion with trypsin or chymotrypsin as described by Liu et al.^48^. The peptides were extracted with acetonitrile containing 0.1% formic acid and vacuum dried. Proteolytic peptides were reconstituted with mobile phase A (2% acetonitrile containing 0.1% formic acid) and then separated on an on-line C18 column (75 μM inner diameter, 360 μM outer diameter, 10 cm, 3 μM C18). Mass spectrometry analysis was carried out on an LTQ-Orbitrap Velos mass spectrometer (Thermo Fisher Scientific, Waltham, MA, USA) operated in data dependent scan mode. Survey scan (m/z 375–1300) was performed at a resolution of 60,000 followed by MS2 scans to fragment the 50 most abundant precursors with collision induced dissociation. The activation time was set at 30 ms, the isolation width was 1.5 amu, the normalized activation energy was 35%, and the activation q was 0.25. Mass spectrometry raw file was searched by Proteome Discovery version 1.3 using MASCOT search engine with percolator against the human ref-sequence protein database (updated on 07–04–2013). Phosphorylation of Ser/Thr and Tyr, acetylation of Lys, mono-methylation and di-methylation of Lys and Arg, and di-glycine modification of Lys (ubiquitination) were used as variable modifications. A filter of 90% peptide confidence was applied according to the Peptide-Prophet and Protein-Prophet parsimony algorithms. Fragment assignment of each modified peptide was subject to manual inspection and validation using the original tandem mass spectra acquired in profile mode using Xcalibur software^16^.

### Statistical analyses

The band intensities of all the immunoblots were quantified by the ImageJ software and presented as means ± SD of triplicate measurements in one experiment representative of three similar ones. All RT-PCR quantitative data were presented as means ± SD of three independent experiments. The statistical significance of normally distributed data was evaluated using the two-tailed unpaired Student's test, and differences were considered significant at *P < 0.05 and **P < 0.01.

## Supplementary Information

Supplementary Information 1.

Supplementary Information 2.

Supplementary Information 3.

Supplementary Information 4.

Supplementary Information 5.

Supplementary Information 6.

Supplementary Information 7.
