# Supplementary material for: LSD1-mediated demethylation of OCT4 safeguards pluripotent stem cells by maintaining the transcription of PORE-motif-containing genes
Source: Sci Rep. 2021 May 13;11:10285. doi: 10.1038/s41598-021-89734-y (PMC8119428; doi:10.1038/s41598-021-89734-y)
Supplement: Supplementary file 7 — Supplementary Information 7. [file 41598_2021_89734_MOESM7_ESM.pdf]

Supplemental Information

LSD1-mediated demethylation of OCT4 safeguards pluripotent stem cells by maintaining the transcription of PORE-motif-containing genes

Songsong Dan, Yuelin Song, Xiaotao Duan, Xiao Pan, Cheng Chen, Shiqi She, Tong Su, Jingchao Li, Xinyu Chen, Yanwen Zhou, Wenjie Chen, Xiaobing Zhang, Xiaoyun Pan, Ying-Jie Wang, and Bo Kang

Inventory of Supplemental Information

Supplemental Figures

Figure S1. The PTM profiles of recombinant OCT4 proteins incubated with whole cell lysates of various human cells.
Figure S2. Mono-methylation of recombinant OCT4 proteins incubated with whole cell lysates of U87 cells via mass spectrum.
Figure S3. MS identification of peptides spanning the methylated OCT4-K222 in 293T and NCCIT cells ectopically-expressed with FLAG-OCT4-6His.
Figure S4. Alignment of LSD1 substrate protein sequence of both histone 3 and non-histones.
Figure S5. Subcellular localization of FLAG-OCT4 proteins ectopically-expressed in 293T and NCCIT cells.
Figure S6. Subcellular localization of FLAG-OCT4 proteins ectopically-expressed in HeLa cells treated with MG-132.
Figure S7. Subcellular localization of endogenous OCT4 proteins in NCCIT cells treated with LSD1 inhibitors.
Figure S8. OCT4 WT and Variants Hydropathy Plot_EMBOSS Pepwindow Online.
Figure S9. PORE(T) genes of OCT4 ChIP binding and CDH13 as one of genes with OCT4 PORE(T) motif down-regulated in shLSD1/ESC cells.
Figure S10. mRNA levels of PORE-like genes in H9 cells transfected with shOCT4-FLAG-OCT4 constructs expressing WT OCT4 or OCT4-K222 mutants.
Figure S11. RNA-seq data showing mRNA levels of PORE(T) genes in NCCIT cells treated with 10 μM RA for 0, 3 or 9 days..
Figure S12. The stemness and differentiation of embryonic carcinoma cells treated with retinoic acid, CHX and LSD1 inhibitors.
Figure S13. Graphical summary.

Supplemental Tables

Table S1. OCT4 PTM sites identified by MS.
Table S2. List of candidate PORE genes and PORE-like genes.
Table S3. Search for PORE-like motifs in stemness and germ layer genes.
Table S4. Candidate PORE gene functions.
Table S5. All RT-PCR primers.
Table S6_Candidate PORE genes bound and regulated by OCT4

Supplemental Experimental Procedures

– Cell culture, Transfection and Treatment
– Antibodies and Reagents
– Plasmids and Viral Infection
– Immunoprecipitation and Immunoblotting
– Immunofluorescence Staining and Co-localization Study
– Electrophoretic Mobility Shift Assay, EMSA
– Quantitative Real-Time PCR, qRT-PCR
– Luciferase Reporter-based OCT4 Transactivity Assay
– Recombinant Protein Expression and Purification
– *In Vitro PTM of Recombinant OCT4 Proteins*
– Mass Spectrometric Identification of PTMs in OCT4 Proteins
– Statistical Analyses

Supplemental References

Uncropped Immunoblots

Figure S1

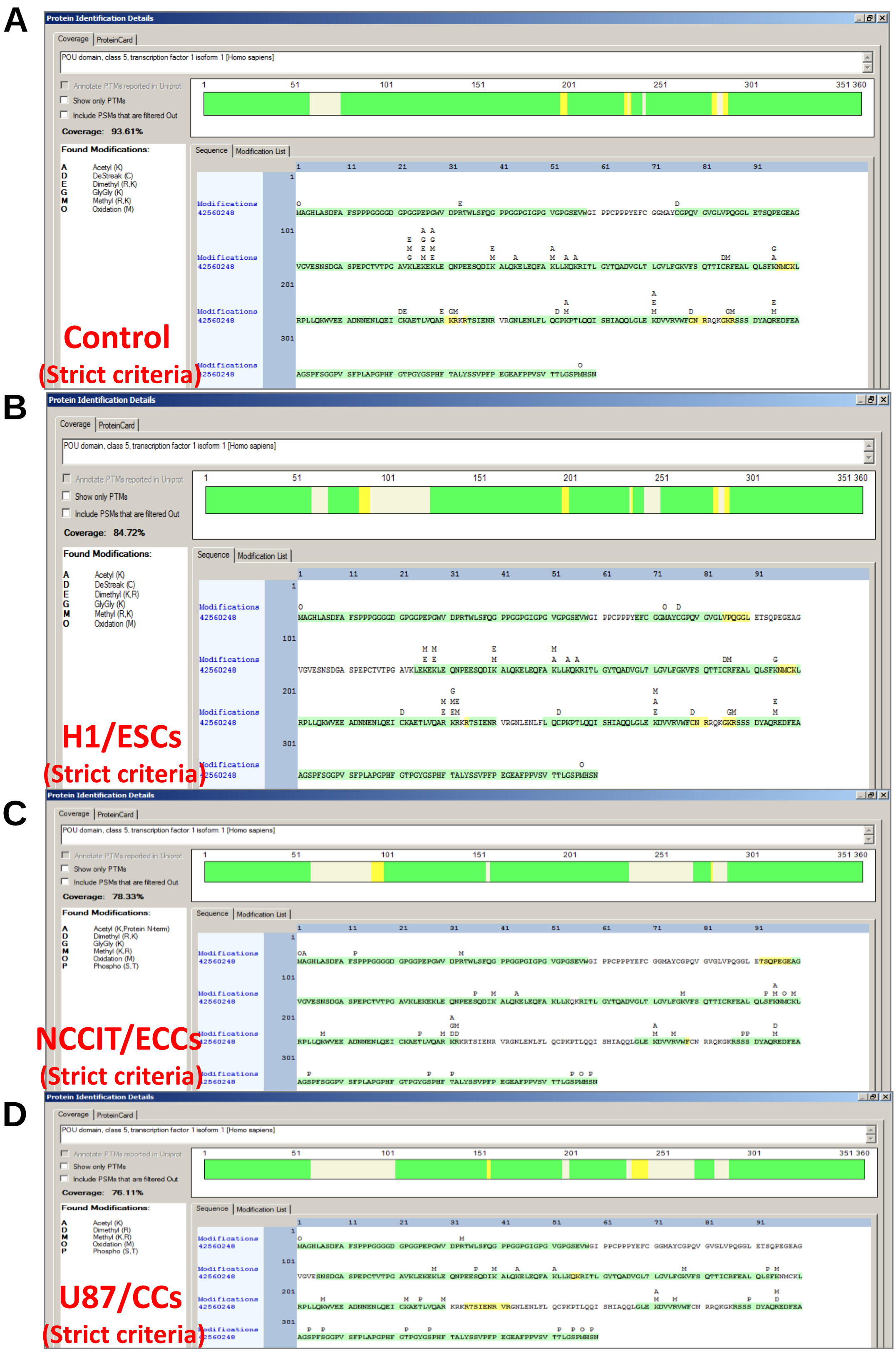

Figure S1. The PTM profiles of recombinant OCT4 proteins incubated with whole cell lysates of various human cells (Control, H1, NCCIT and U87).

Figure S2

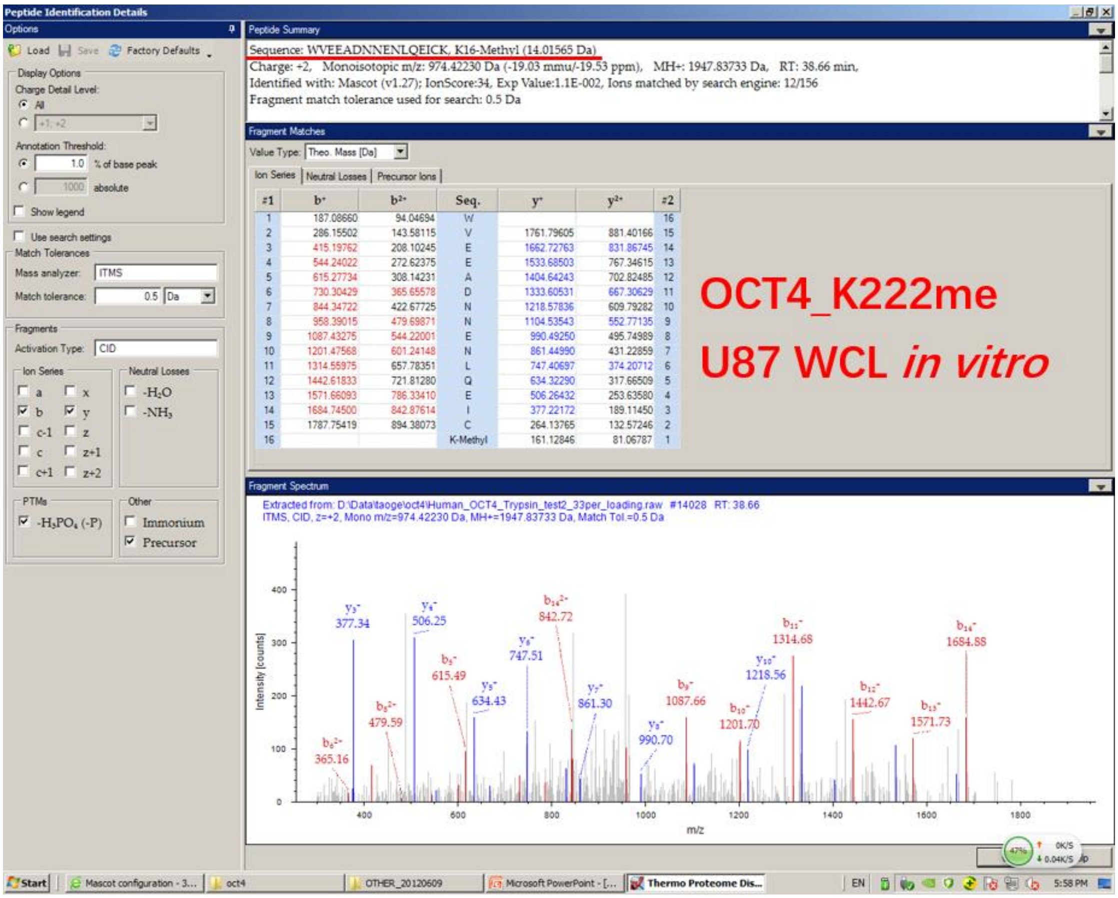

Figure S2. Mono-methylation of recombinant OCT4 proteins incubated with whole cell lysates of U87 cells via mass spectrum.

Figure S3

A

PTMs	293T cells_IP *in vivo*	NCCIT cells_IP *in vivo*
Mono-Me	K222me	K222me
Di-Me	K222me2	K222me2

B

keratin, type II cytoskeletal 5 [Homo sapiens]	65.41	18.64	9	6	11	11	4.021E7	590	62.3	7.74
eukaryotic translation initiation factor 3 subunit E [Homo sapie	64.48	36.18	1	11	14	17	3.159E7	445	52.2	6.04
POU domain, class 5, transcription factor 1 isoform 1	64.33	21.11	3	9	11	29	5.557E8	360	38.5	5.94
A4	Sequence	# PSMs	# Proteins	# Protein Groups	Protein Group Accessions	Modifications	ΔCn	Area	q-Value	PEP
293T cells High	WVEEADNNENLQEIck	5	3	1	42560248	C15(Carbamidomethyl); K16(I	0.0000	1.442E9	0	0.00004632
High	VFSQTTICR	1	2	1	42560248		0.0000	2.024E7	0	0.00512
FLAG-OCT4-6His High	WVEEADNNENLQEIcK	1	3	1	42560248	C15(DeStreak)	0.0000	0.000E0	0	0.00003561
High	EKLEQNPEESQDIKALQK	1	1	1	42560248		0.0000	1.450E7	0	0.001446
High	ALQKELEQFAKLLK	1	2	1	42560248		0.0000	0.000E0	0	0.002812
High	WVEEADNNENLQEIck	1	3	1	42560248	C15(Carbamidomethyl); K16(Di	0.0000	6.123E6	0	0.00004545
High	WVEEADNNENLQEIck	1	3	1	42560248	C15(DeStreak); K16(Dimeth	0.0000	0.000E0	0	0.004069
High	EKLEQNPEESQDIK	1	1	1	42560248		0.0000	0.000E0	0.003	0.01499
High	AETLVQAR	4	2	1	42560248		0.0000	4.314E6	0.003	0.007252
High	ELEQFAK	2	2	1	42560248		0.0000	0.000E0	0.003	0.01089
High	SSSDYAQR	1	3	1	42560248		0.0000	2.046E8	0.003	0.007651
High	LRPLLQK	7	3	1	42560248		0.0000	0.000E0	0.009	0.08267
Low	LEQNPEESQDIK	1	1	1	42560248		0.0000	0.000E0	0.054	0.343
Low	LEQNPEESQDIk	1	1	1	42560248	K12(Dimethyl)	0.0000	7.380E6	0.076	0.4525
Low	LEQNPEESQDIkALQK	1	1	1	42560248	K12(Dimethyl)	0.0000	2.089E6	0.618	1

C

keratin, type I cytoskeletal 10 [Homo sapiens]	138.79	36.13	1	12	16	19	9.312E7	584	58.8	5.21
serpin H1 precursor [Homo sapiens]	137.58	45.69	1	15	17	25	1.109E8	418	46.4	8.69
POU domain, class 5, transcription factor 1 isoform 1	136.20	30.00	7	12	12	62	1.321E9	360	38.5	5.94
A4	Sequence	# PSMs	# Proteins	# Protein Groups	Protein Group Accessions	Modifications	ΔCn	Area	q-Value	PEP
NCCIT cells High	WVEEADNNENLQEIck	7	3	1	42560248	(Carbamidomethyl); K16(Met	0.0000	8.913E6	0	0.002219
High	FEALQLSFK	6	7	1	42560248		0.0000	1.365E7	0	0.01468
FLAG-OCT4-6His High	LEKEKLEQNPEESQDIK	2	1	1	42560248		0.0000	4.612E7	0	0.007398
High	AETLVQAR	7	2	1	42560248		0.0000	5.227E6	0	0.02446
High	WVEEADNNENLQEIcK	3	3	1	42560248	C15(DeStreak)	0.0000	8.099E8	0	0.001552
High	WVEEADNNENLQEIck	1	3	1	42560248	Carbamidomethyl); K16(Dime	0.0000	4.759E6	0	0.01299
High	LEQNPEESQDIK	4	1	1	42560248		0.0000	1.344E9	0.004	0.03293
High	EKLEQNPEESQDIK	4	1	1	42560248		0.0000	1.808E9	0.004	0.0708
High	ITLGYTQADVGLTLGVLFGK	1	1	1	42560248		0.0000	0.000E0	0.004	0.04435
High	VFSQTTICR	1	2	1	42560248		0.0000	2.358E7	0.006	0.1187
High	SSSDYAQR	2	3	1	42560248		0.0000	3.545E7	0.006	0.08634
High	LRPLLQK	20	3	1	42560248		0.0000	3.532E6	0.006	0.1176
Medium	ELEQFAK	3	2	1	42560248		0.0000	5.766E5	0.013	0.1811
Medium	ALQKELEQFAKLLK	1	2	1	42560248		0.0000	1.754E7	0.013	0.1846

Figure S3. MS identification of peptides spanning the methylated OCT4-K222 in 293T and NCCIT cells ectopically-expressed with FLAG-OCT4-6His. (A) Methylation sites of exogenous OCT4 ectopically-expressed in 293T and NCCIT cells. FLAG-OCT4-6His proteins were pulled down via Ni-NTA agarose resin, and subjected to MS analysis. (B) and (C) MS identification of peptides spanning the methylated OCT4-K222 in 293T and NCCIT cells.

Figure S4

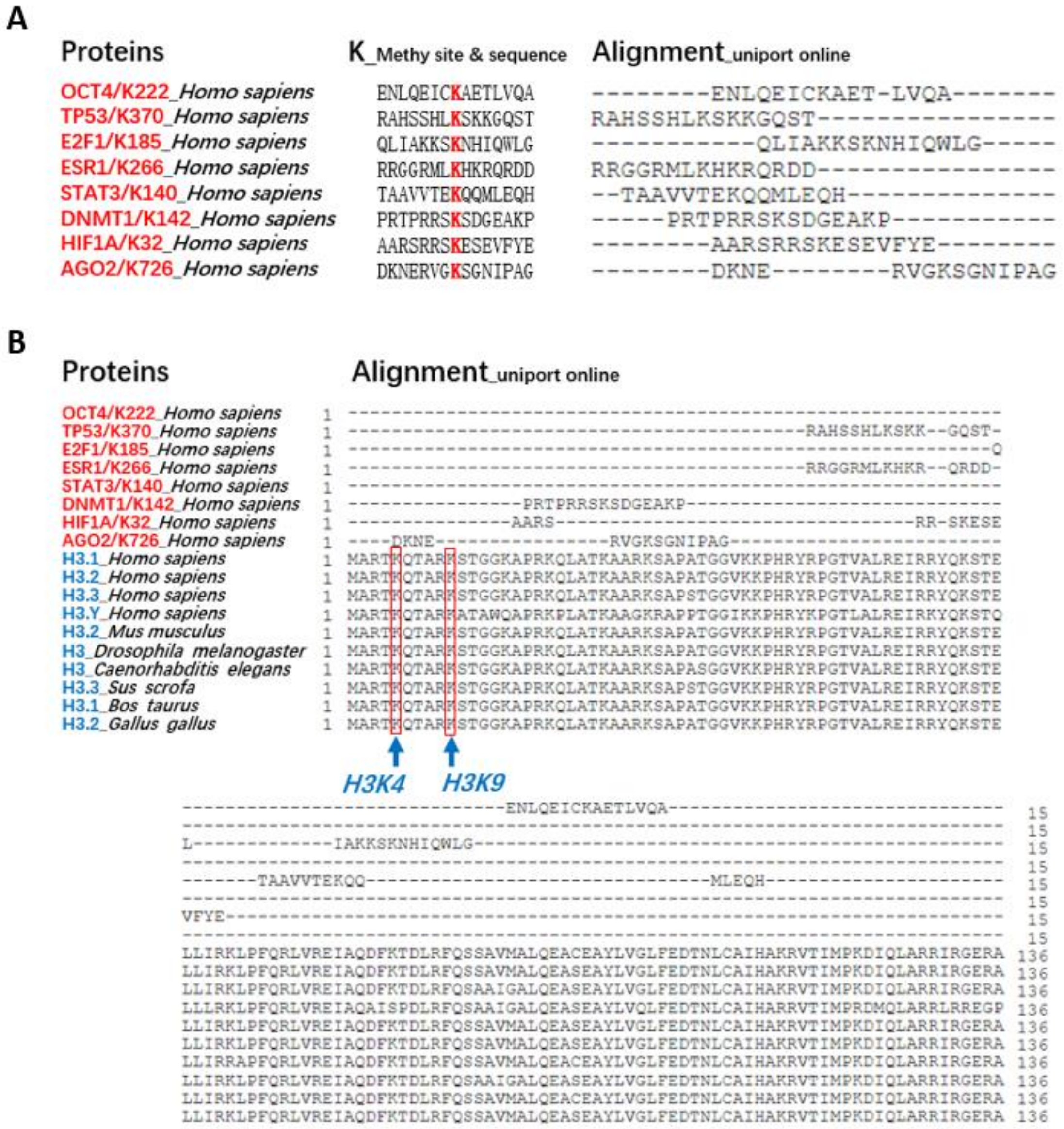

Figure S4. Alignment of LSD1 substrate protein sequence of both histone 3 and non-histones. (A) Alignment of protein sequence of non-histone LSD1 substrate proteins. (B) Alignment of LSD1 substrate protein sequence of both histone 3 and non-histones.

Figure S5

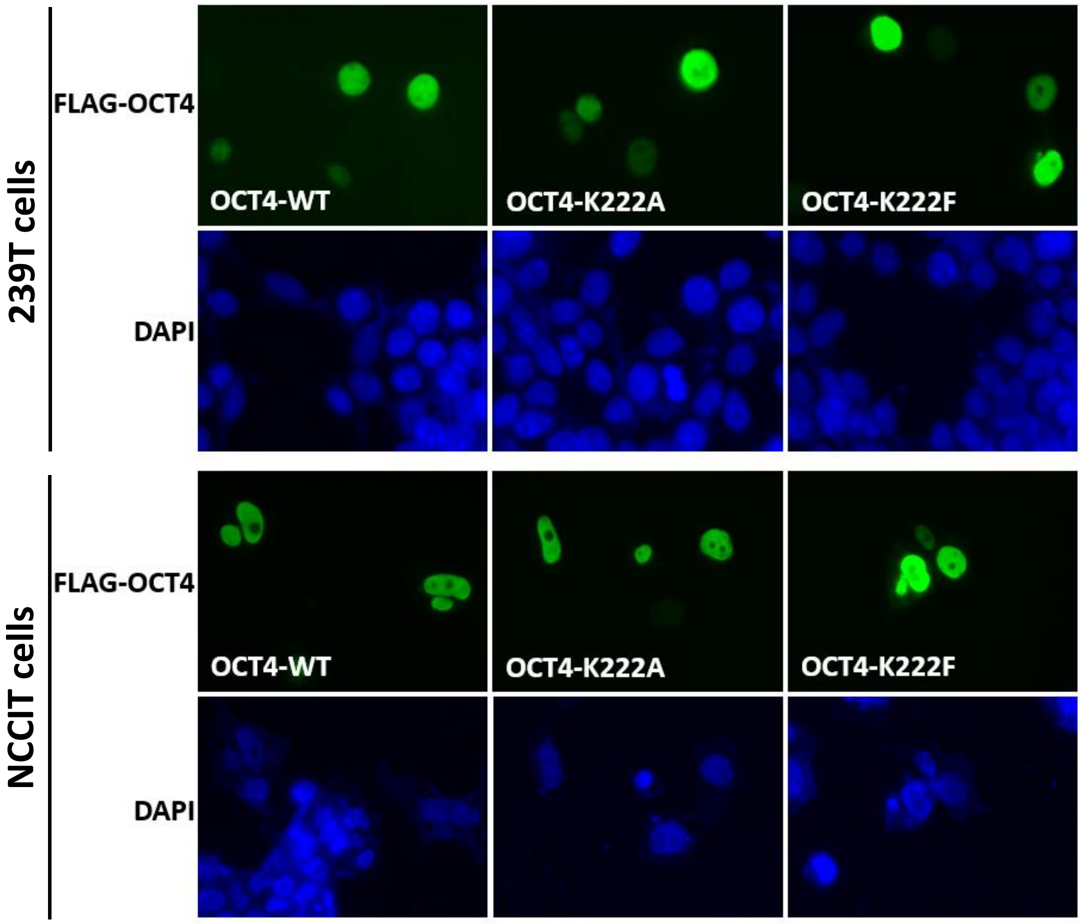

Figure S5. Subcellular localization of FLAG-OCT4 proteins ectopically-expressed in 293T and NCCIT cells. 293T and NCCIT cells were transfected with shOCT4-FLAG-OCT4 (WT and variants). After 72 hours, the localization of OCT4 was examined by immunofluorescence microscopy using anti-DYKDDDDK-tag (FLAG) mouse antibody.

Figure S6

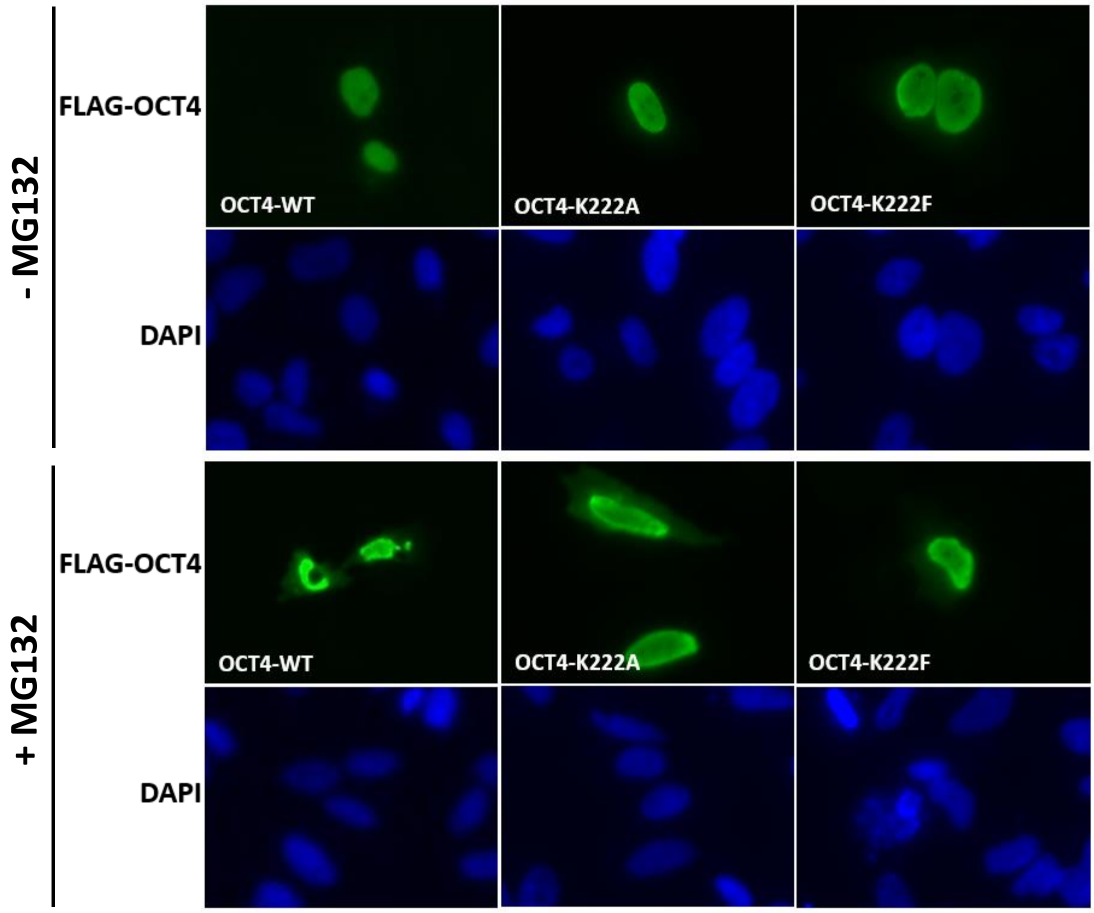

Figure S6. Subcellular localization of FLAG-OCT4 proteins ectopically-expressed in HeLa cells treated with MG-132. 293T cells were transfected with shOCT4-FLAG-OCT4 (WT and variants) for 72 hours and treated with 5 μM MG132 for 12 hours. The localization of OCT4 was examined by immunofluorescence microscopy using anti-DYKDDDDK-tag (FLAG) mouse antibody.

Figure S7

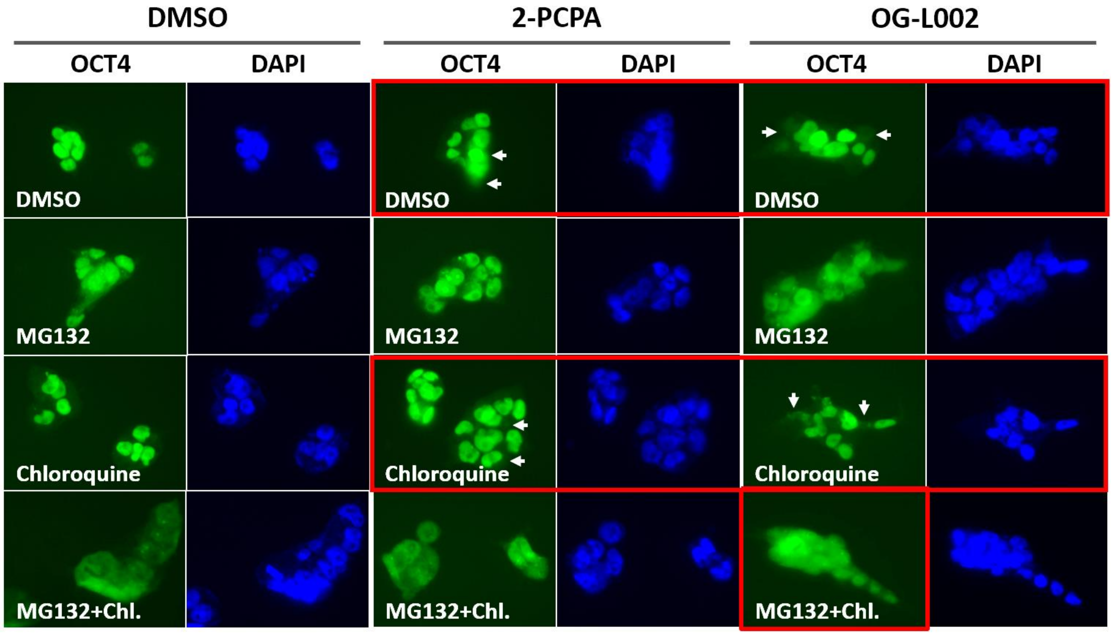

Figure S7. Subcellular localization of endogenous OCT4 proteins in NCCIT cells treated with LSD1 inhibitors. NCCIT cells were treated with 5 μM MG132 together with LSD1 inhibitors for 12 hours. The localization of OCT4 was examined by immunofluorescence microscopy using anti-Oct-3/4 (C-10) mouse antibody.

Figure S8

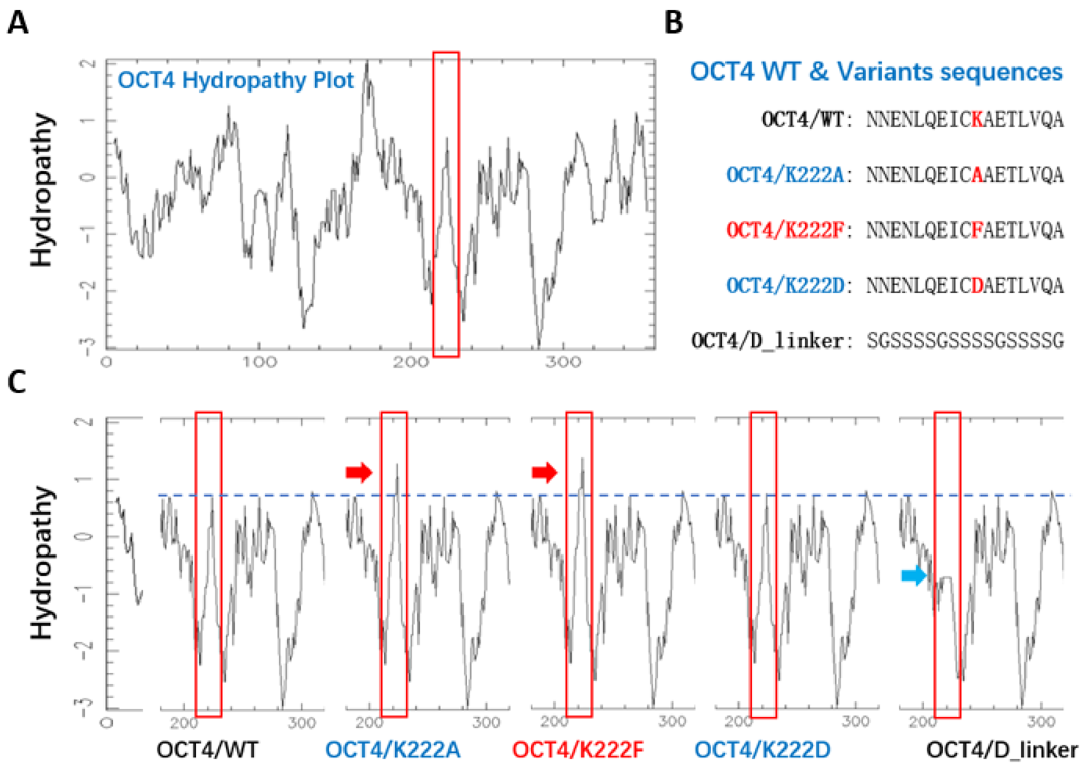

Figure S8. OCT4 WT and Variants Hydropathy Plot_EMBOSS Pepwindow Online. (A) Hydropathy Plot of full-length wild type (WT) OCT4 protein. (B) The linker sequence of OCT4 WT and its variants. (C) Comparing the predicted hydrophobicity of the linker regions between OCT4 WT and its variants.

Figure S9

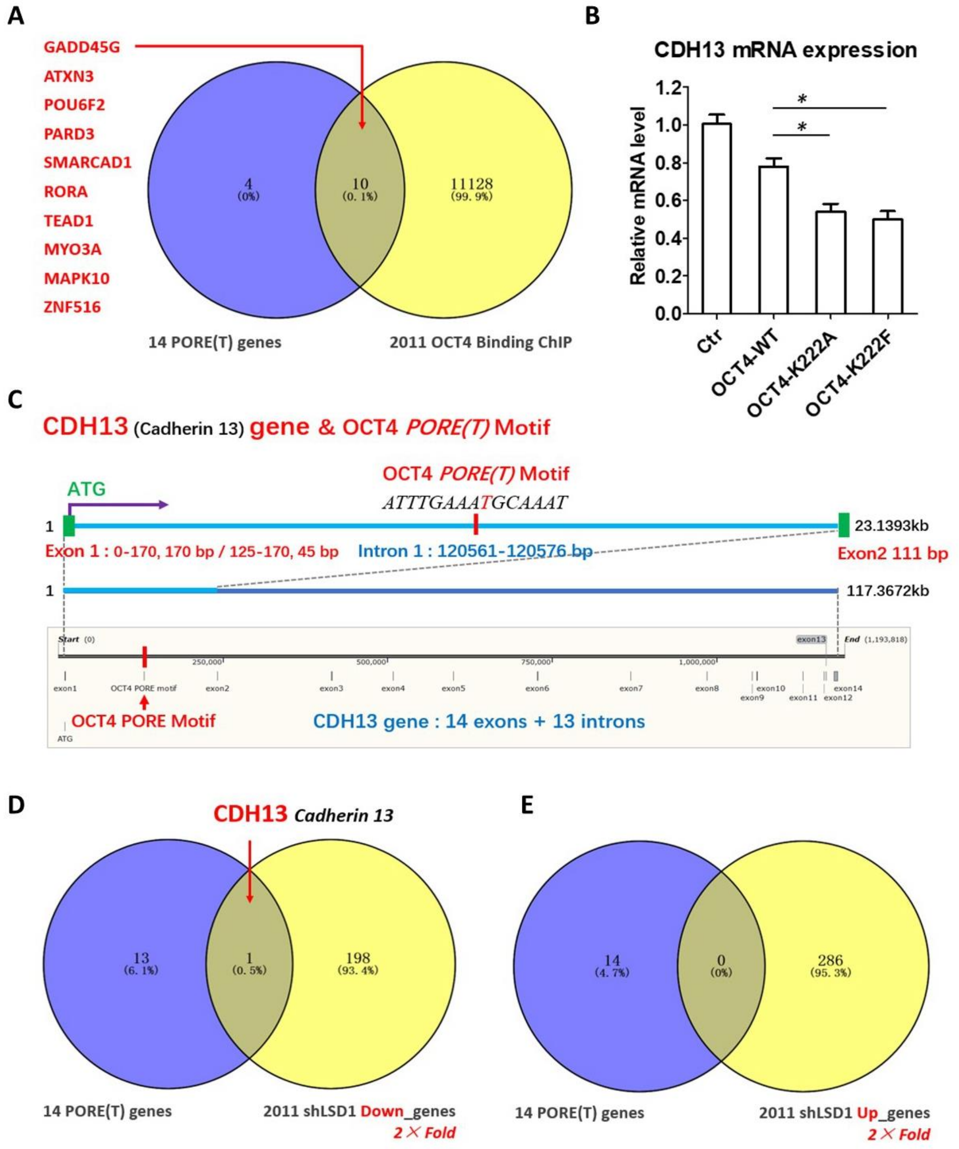

Figure S9. PORE(T) genes of OCT4 ChIP binding and CDH13 as one of genes with OCT4 PORE(T) motif down-regulated in shLSD1/ESC cells. (A) 10 of 14 PORE(T) genes were found in OCT4 ChIP binding genes. (B) The relative mRNA levels of CDH13 determined by qRT-PCR. (C) The location of OCT4 PORE(T) motif on CDH13 gene. (D) CDH13 is the only one gene containing PORE(T) that has over 2 fold down-regulation in shLSD1-ESCs published by 2011 Adamo, A. et al. (E) PORE(T)-containing genes that have over 2 fold up-regulation in shLSD1-ESCs published by 2011 Adamo, A. et al. Results shown in (B) were presented as means ±S.D. of triplicate measurements from single experiment. Two-tailed unpaired Student's t tests were used for statistical analyses. *P < 0.05 and **P < 0.01.

Figure S10

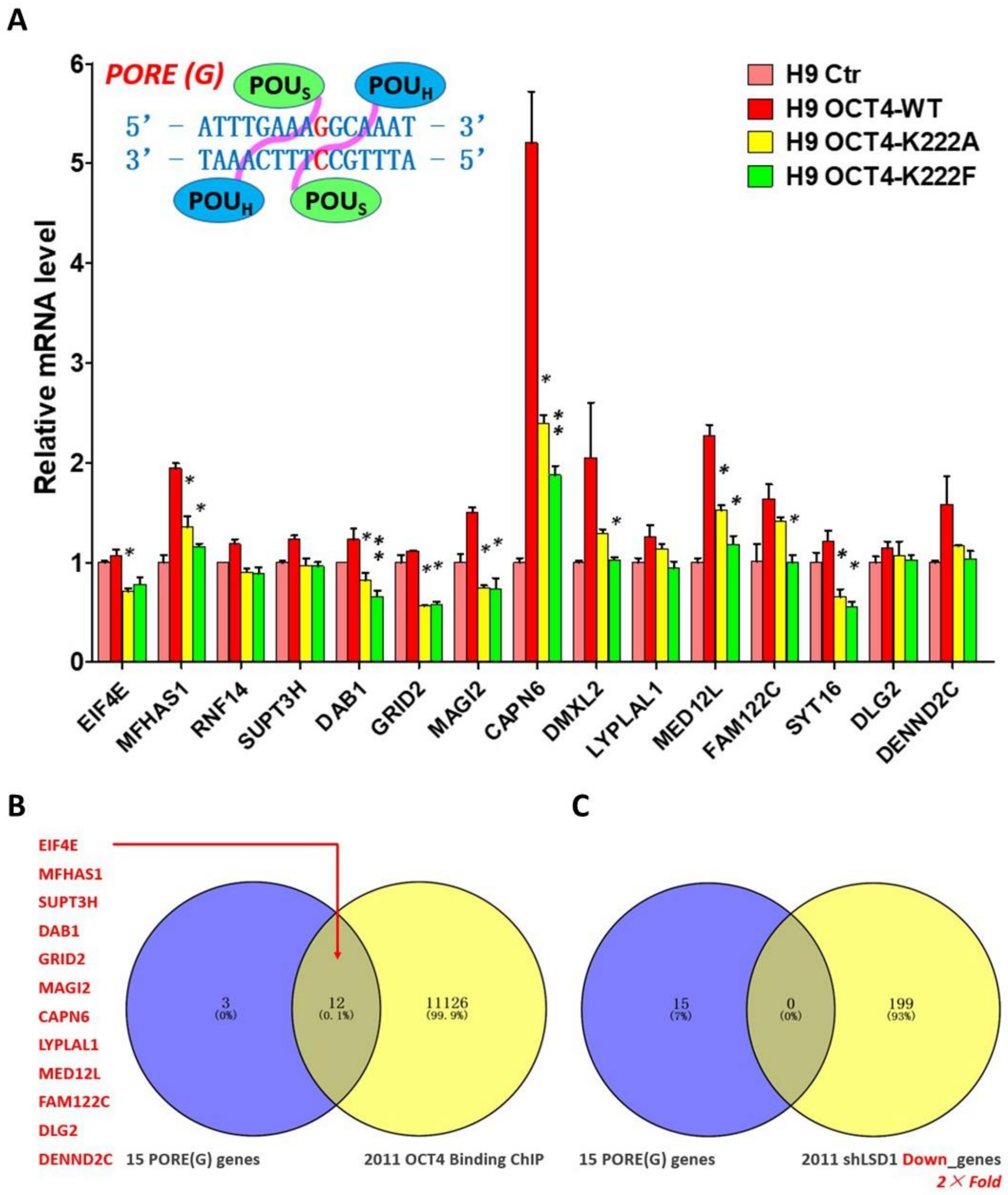

Figure S10. mRNA levels of PORE-like genes in H9 cells transfected with shOCT4-FLAG-OCT4 constructs expressing WT OCT4 or OCT4-K222 mutants. (A) H9 cells were transfected with shOCT4-FLAG-OCT4 (WT and variants) constructs with the empty vector as a control. 72 hours later, cells were harvested and the relative mRNA levels of the PORE(G) genes were determined by qRT-PCR. (B) 11 of 15 PORE(G) genes were found in OCT4 ChIP binding genes. (C) PORE(G) genes that have over 2 fold down-regulation in shLSD1-ESCs published by 2011 Adamo, A. et al. Results shown in (A) were presented as means ±S.D. of triplicate measurements from single experiment. Two-tailed unpaired Student's t tests were used for statistical analyses. *P < 0.05 and **P < 0.01.

Figure S11

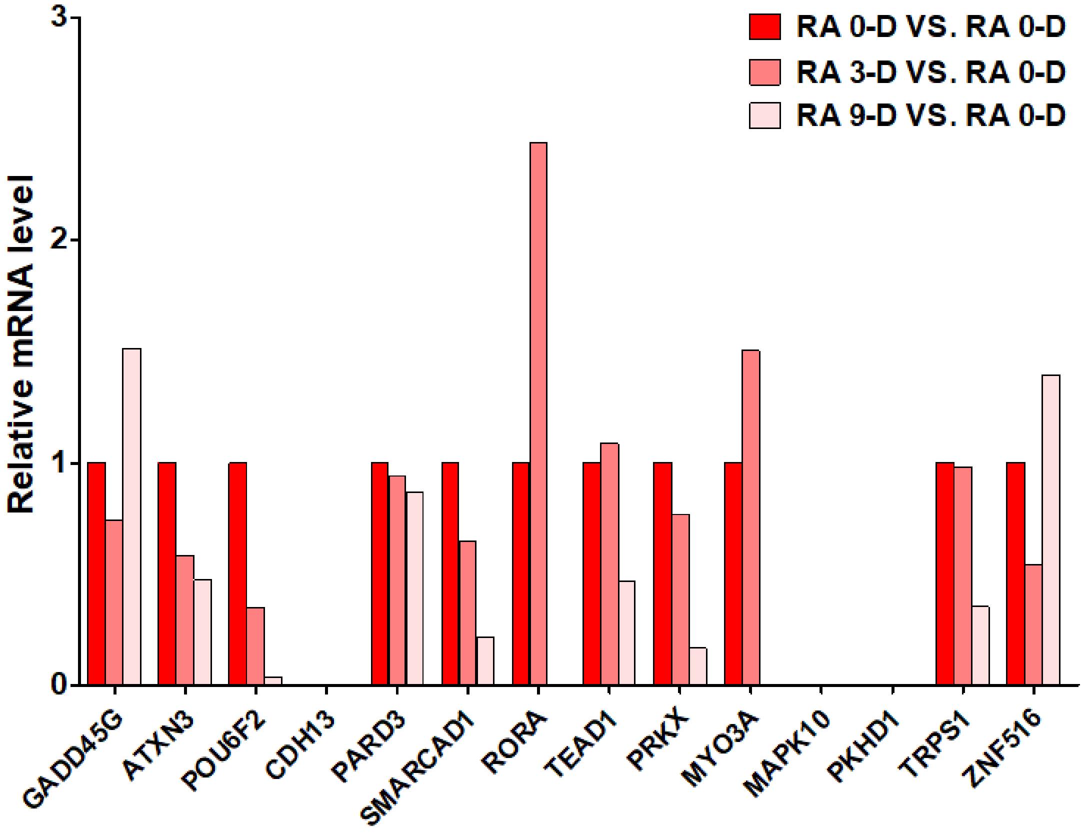

Figure S11. RNA-seq data showing mRNA levels of PORE(T) genes in NCCIT cells treated with 10 μM RA for 0, 3 or 9 days.

Figure S12

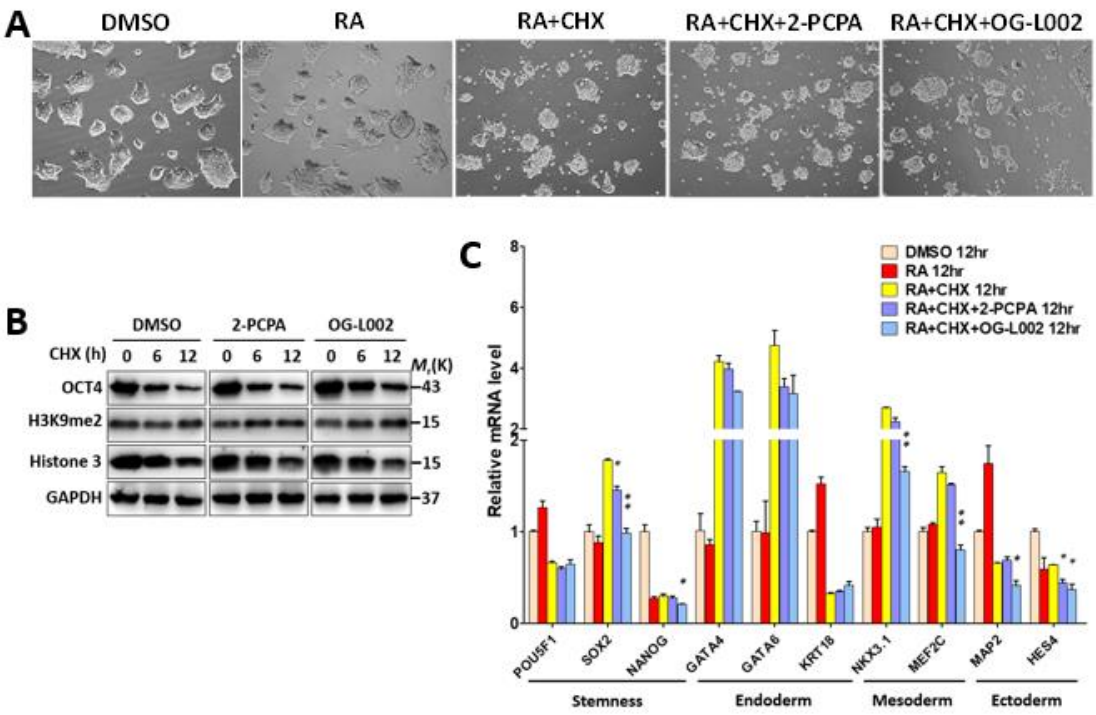

Figure S12. The stemness and differentiation of embryonic carcinoma cells treated with retinoic acid, CHX and LSD1 inhibitors. (A) The morphology of NCCIT cells pre-treated with 10 μM retinoic acid (RA) alone or in combination with 100 μM 2-PCPA or 50 μM OG-L002 for 1 hour, followed by addition of 20 μg/ml CHX and further incubation for 12 hours. (B) NCCIT cells were treated with 100 μM 2-PCPA or 50 μM OG-L002 for 1 hour, followed by addition of 20 μg/ml CHX and further incubation for up to 12 hours. Whole cell lysates were immunoblotted with the indicated antibodies. (C) The relative mRNA levels of the stemness genes and lineage-specific marker genes in NCCIT cells treated as in (A). Results shown in (C) were presented as means ±S.D. of triplicate measurements from single experiment. Two-tailed unpaired Student's t tests were used for statistical analyses. *P < 0.05 and **P < 0.01.

Figure S13

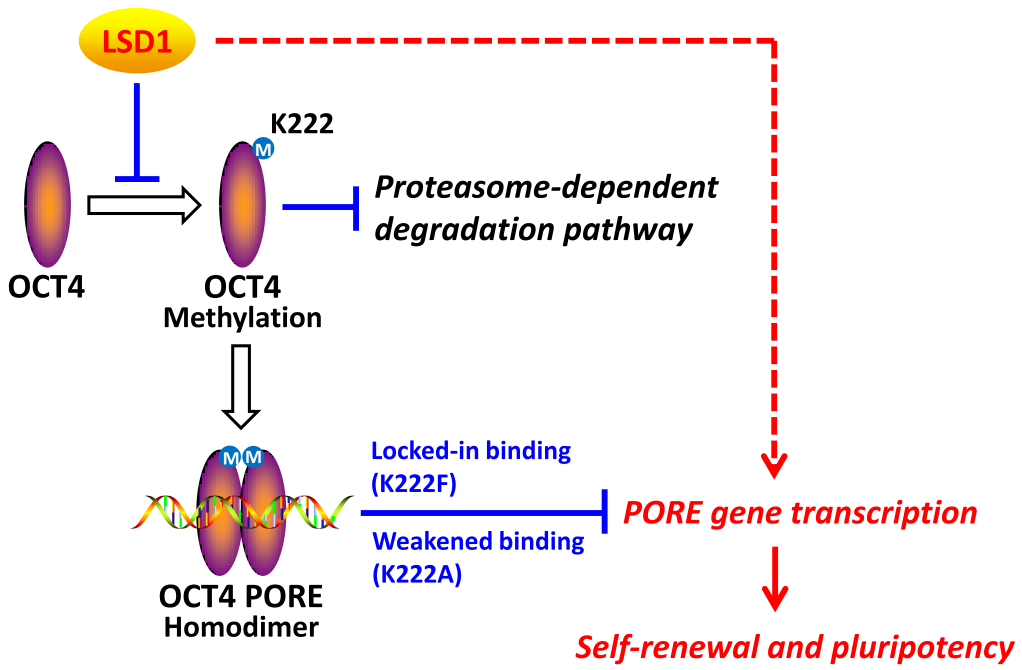

Figure S13. Graphical summary.

Supplemental Experimental Procedures

Cell culture, Transfection and Treatment

The sources and culture conditions for 293T, HeLa, HepG2, U87, U251, HT29, LO2, HUVEC, MCF7, H1, H9 and NCCIT cells were described in detail previously (Chen et al., 2012; Cheng et al., 2015; Dan et al., 2015). All cells were maintained in a 37 ℃ incubator with 5% CO2. H1 and H9 cells were cultured in mTeSR1 medium from STEMCELL (catalog no. 85850). Neural stem cells (NSCs) derived from H1 and H9 cells were cultured in Neural Progenitor Medium from STEMCELL (catalog no. 05833). 293T, HeLa and U87 cells were transfected with GenEscort II Transfection Reagent reagents (catalog no. WIS 2100) as described from WISGEN. H1 and H9 cells were transfected with Lipofectamine 3000 Transfection Reagent (catalog no. L3000015) as described in the instructions provide by Thermo-Fisher. In some experiments, cells were treated with 20 μg/ml CHX, 5 μM MG-132, 200 μM Chloroquine, 10 μM Retinoic acid (RA), 100 μM Tranylcypromine (2-PCPA) HCl and 50 μM OG-L002 (DMSO as the vehicle) either individually or in combination.

Antibodies and Reagents

The anti-Oct-3/4 (C-10) mouse antibody (catalog no.sc-5279), anti-Oct-3/4 (H-134) rabbit antibody (catalog no. sc-9081), anti-LSD1 (B-9) antibody (catalog no. sc-271720) and anti-Histone H3 (C-16) antibody (catalog no. sc-8654) were purchased from Santa Cruz Biotechnology. Anti-DYKDDDDK-tag mouse antibody (catalog no. A00187) and anti-DYKDDDDK-tag rabbit antibody (catalog no. A01870), anti-His antibody (catalog no. A00186), anti-GAPDH (HRP) antibody (catalog no. A00192), goat anti-mouse IgG antibody (H&L) [HRP] (catalog no. A00160) and goat anti-rabbit IgG antibody (H&L) [HRP] (catalog no. A00098) were from GenScript. Anti-Myc-tag mouse antibody (catalog no. AM926), anti-Myc-tag rabbit antibody (catalog no. AM933), di-methyl-histone H3 (Lys4) antibody (catalog no. AF5653) and di-methyl-histone H3 (Lys9) antibody (catalog no. AF2314) were from Beyotime. Anti-pan-methylated lysine(di-/mono-methyl) antibody (catalog no. ab23366) was from Abcam. Anti-pT235 rabbit antibody was described in detail previously (Chen et al., 2012; Dan et al., 2015). Anti-mouse IgG DyLight 488 (catalog no. 715-485-150) and anti-mouse IgG DyLight 594 (catalog no. 715-515-150) were from Jackson ImmunoResearch. Cycloheximide (catalog no. 239764) and MG-132 (catalog no. 474790) were purchased from Calbiochem. Anti-FLAG M2 Magnetic Beads (catalog no. M8823-1ML) was from Sigma-Aldrich. Pierce protein A/G agarose (catalog no. 20421) was from Thermo-Fisher. Ni-NTA agarose resin was from QIAGEN (catalog no. 30310). Chloroquine (catalog no. C6569) was from Sangon Biotech. Retinoic acid (RA) (catalog no. 302-79-4) was from Sigma-Aldrich. Tranylcypromine (2-PCPA) HCl (catalog no. S4246) and OG-L002 (catalog no. S7237) were from Selleck.

Plasmids and Viral Infection

The prokaryotic human His-OCT4 and His-SOX2 were generated by PCR and subcloned into the pET28a vector via EcoRI and SacI sites, and His-LSD1 (852 AA) was generated via NheI and XhoI sites. Lentiviral vector was constructed targeting the 3’-UTR of human POU5F1 mRNA to knock down the endogenous OCT4 while expressing the FLAG-tagged exogenous

OCT4 WT (referred to as “shOCT4+FLAG-POU5F1-WT”) was described previously (Lin et al., 2012), which was applied to infect and transfect the cells for 48-96 hours as described in detail previously(Lin et al., 2012). The OCT4 variants (K222A, K222R, K222F and K222D) were generated from OCT4 WT (His-OCT4 or shOCT4+POU5F1) plasmids by site-directed mutagenesis and applied to the experiments. The FLAG-OCT4-6His portion was subcloned into the EcoRI and XbaI sites of the pLKO.1-TRC-shOCT4-FLAG-OCT4 plasmid (Lin et al., 2012).

Immunoprecipitation and Immunoblotting

Immunoprecipitation and immunoblotting were conducted as described previously (Dan et al., 2015; Lin et al., 2012). 1 mg whole cell lysates (293T and NCCIT) were incubated with 50 µl anti-FLAG M2 magnetic beads or 50 µl Pierce™ protein A/G agarose over 8 hours at 4℃ respectively. 1 mg of whole cell lysates (U87, NCCIT and H1) was incubated with 100 µl of Ni-NTA agarose resin over 2 hours at 4℃ respectively. Immunoblotting and Coomassie Brilliant Blue staining were performed after SDS-PAGE.

Immunofluorescence Staining and Co-localization Study

Immunofluorescence staining was conducted as described previously (Lin et al., 2012). Briefly, anti-OCT4 and anti-LSD1 antibodies were applied for immunofluorescence staining and their co-localization in NCCIT cells was determined by confocal microscopy (Carl Zeiss LSM-510).

Electrophoretic Mobility Shift Assay, EMSA

EMSAs were carried out using a LightShift chemiluminescent EMSA kit (Thermo-Fisher, catalog no. 20148) as instructed by the manufacturer. Briefly, a 20 µl reaction mixture containing 1.5 µg purified recombinant His-OCT4 (WT and variants), 0.5 µg purified recombinant His-SOX2, 50 nM 5’-biotin end-labeled dsDNA probes (NANOG-SORE, OCT4-SORE, PORE, MORE) and 1 µl Poly (dl·dC) in 1×Loading buffer was incubated for 30 min at 25℃. The sequences of the probes were as follows:

NANOG-SORE probe,

Sense:5’-biotin-GTCTGGGTTACTCTGCAGCTACTTTTGCATTACAATGGCCTTGGTGA
GACTGGTAGACG-3’

Anti-sense:5’-CGTCTACCAGTCTCACCAAGGCCATTGTAATGCAAAAGTAGCTGCAG
AGTAACCCAGAC-3’

OCT4 SORE probe,

Sense:5’-biotin-CCGTCTTCTTGGCAGACAGCAGAGAGATGCATGACAAAGGTGCCG
TGATGGTTCTGTCC-3’

Anti-sense:5’-GGACAGAACCATCACGGCACCTTTGTCATGCATCTCTCTGCTGTCTGC
CAAGAAGACGG-3’

OCT4 PORE probe,

Sense:5’-biotin-TATACTAAGCAATTCTTCATTGATTTGAAATGCAAATTTGACTGGGC
ACCCTGTATCTT-3’

Anti-sense:5’-AAGATACAGGGTGCCCAGTCAAATTTGCATTTCAAATCAATGAAGAA
TTGCTTAGTATA-3’

OCT4 MORE probe,
Sense:5'-biotin-TGTGAAATACCCTGCCTCATGCATATGCAAATAACCTGAGGTCTTCTGAGATAAATATA-3'
Anti-sense:5'-TATATTTATCTCAGAAGACCTCAGGTTATTTGCATATGCATGAGGCAGGGTATTTCACA-3'

Quantitative Real-Time PCR, qRT-PCR

Quantitative real time PCR analysis and primers used for lineage marker quantitation were as described previously (Lin et al., 2012). Firstly, total RNA was extracted by RNAiso Plus (TaKaRa, catalog no. 9109). Secondly, cDNA was synthesized using PrimeScript RT reagent kit with gDNA eraser (TaKaRa, catalog no. RR047A) according to the manufacturer's instructions. Thirdly, qRT-PCR was performed using the iTaq Universal SYBR Green Supermix (Bio-Rad, catalog no. 1721-5124) in an ABI 7500 Real-Time PCR instrument. All the PCR amplifications were performed in triplicates and repeated in three independent experiments. The relative quantities of mRNAs were normalized by the mRNA levels of the housekeeping gene PBGD (HMBS). The sequences of all RT-PCR primers were listed in Table S5.

Luciferase Reporter-based OCT4 Transactivity Assay

The pGL6-TA vector (Beyotime, catalog no. D2105) was inserted with three tandem repeats of one of the OCT4 binding motifs (3SORE, 3PORE, 3MORE and 3MONO) via XhoI and HindIII sites. The resulting constructs together with WT OCT4 or OCT4 variant plasmids were co-transfected into HeLa cells, and the luciferase mRNA expression levels driven by the OCT4 binding motifs were determined by qRT-PCR and normalized by the GAPDH mRNA levels. The sequences of the three tandem repeats of OCT4 binding motifs were as follows:
3SORE, 5'-TTTTGCATTACAATGTTTTGCATTACAATGTTTTGCATTACAATG
3PORE, 5'-ATTTGAAATGCAAATATTTGAAATGCAAATATTTGAAATGCAAAT
3MORE, 5'-ATGCATATGCAAATATGCATATGCAAATATGCATATGCAAAT
3MONO, 5'-ATTTGCATATTTGCATATTTGCAT

Recombinant Protein Expression and Purification

The His-tagged human OCT4 (WT and variants) and SOX2 constructs were transformed into Rosseta E. coli strains and the purification procedures were as described previously (Lin et al., 2012). After sonication, the 15 ml E. coli lysate was centrifuged at 12,000g for 10 min at 4℃ and the supernatants were mixed with 1 ml of 50% (w/v) slurry of Ni-NTA Agarose resin/beads incubated at 4℃ on a rotary shaker for 2 hours. The mixture was then centrifuged at 800 g for 2 min and the supernatant was discarded. The saving beads were washed with lysis buffer containing 50 mM imidazole and the proteins binding on Ni–NTA beads were used for subsequent in vitro PTMs assay (prokaryotic proteins eluted with the same buffer containing 200 mM imidazole for EMSA experiments).

In Vitro PTM of Recombinant OCT4 Proteins

An aliquot (5 μg) of purified recombinant His-OCT4 protein binding to the Ni–NTA beads (100 μl) was incubated with 1 mg of the whole lysate derived from NCCIT, H1 or U87 cells

at 30℃ for 1 hour in 1 ml PMA buffer (50 mM Tris–HCl, pH 7.4, 50 mM KCl, 5 mM $MgCl_2$, 0.5% NP-40, 25 mM imidazole, 5 mM ATP, 100 μM S-adenosyl methionine, 100 μM acetyl-CoA, 1% EDTA-free protease inhibitor cocktail and phosphatase inhibitor cocktail) respectively. 2 μg purified recombinant His-LSD1 protein were added in the demethylation array. The His-OCT4 conjugated beads were sedimented by centrifugation at 800g for 2 min, washed three times with ice-cold PMA washing buffer (50 mM Tris–HCl, pH 7.4, 50 mM KCl, 5 mM $MgCl_2$, 0.5% NP-40, 50 mM imidazole), eluted and denatured in SDS–PAGE sample loading buffer by heating at 100℃ for 5 min. Pooled samples (10-20 μg) were loaded and separated by SDS–PAGE and stained with Coomassie brilliant blue R250. After de-staining, the OCT4 bands (with a molecular weight of 45 kDa) were excised and analyzed by mass spectrometry.

Mass Spectrometric Identification of PTMs in OCT4 Proteins

OCT4 samples were subjected to overnight digestion with trypsin or chymotrypsin as described by Liu et al. (Liu et al., 2013). The peptides were extracted with acetonitrile containing 0.1% formic acid and vacuum dried. Proteolytic peptides were reconstituted with mobile phase A (2% acetonitrile containing 0.1% formic acid) and then separated on an on-line C18 column (75 μM inner diameter, 360 μM outer diameter, 10 cm, 3 μM C18). Mass spectrometry analysis was carried out on an LTQ-Orbitrap Velos mass spectrometer (Thermo Fisher Scientific, Waltham, MA, USA) operated in data dependent scan mode. Survey scan (m/z 375–1300) was performed at a resolution of 60,000 followed by MS2 scans to fragment the 50 most abundant precursors with collision induced dissociation. The activation time was set at 30 ms, the isolation width was 1.5 amu, the normalized activation energy was 35%, and the activation q was 0.25. Mass spectrometry raw file was searched by Proteome Discovery version 1.3 using MASCOT search engine with percolator against the human ref-sequence protein database (updated on 07-04-2013). Phosphorylation of Ser/Thr and Tyr, acetylation of Lys, mono-methylation and di-methylation of Lys and Arg, and di-glycine modification of Lys (ubiquitination) were used as variable modifications. A filter of 90% peptide confidence was applied according to the Peptide-Prophet and Protein-Prophet parsimony algorithms. Fragment assignment of each modified peptide was subject to manual inspection and validation using the original tandem mass spectra acquired in profile mode using Xcalibur software.

Statistical Analyses

The band intensities of all the immunoblots were quantified by the ImageJ software and presented as means ±SD of triplicate measurements in one experiment representative of three similar ones. All RT-PCR quantitative data were presented as means ±SD of three independent experiments. The statistical significance of compared measurements was evaluated using the two-tailed unpaired Student’s t test and the differences were considered significant at $*P < 0.05$ and $**P < 0.01$.

Supplemental References

Chen, X., Yin, Z., Chen, J.L., Shen, W.L., Liu, H.H., Tang, Q.M., Fang, Z., Lu, L.R., Ji, J., and Ouyang, H.W. (2012). Force and scleraxis synergistically promote the commitment of human ES cells derived MSCs to tenocytes. Sci Rep *2*, 977.

Cheng, J., Li, W., Kang, B., Zhou, Y., Song, J., Dan, S., Yang, Y., Zhang, X., Li, J., Yin, S., *et al.* (2015). Tryptophan derivatives regulate the transcription of Oct4 in stem-like cancer cells. Nat Commun *6*, 7209.

Dan, S., Kang, B., Duan, X., and Wang, Y.J. (2015). A cell-free system toward deciphering the post-translational modification barcodes of Oct4 in different cellular contexts. Biochem Biophys Res Commun *456*, 714-720.

Lin, Y., Yang, Y., Li, W., Chen, Q., Li, J., Pan, X., Zhou, L., Liu, C., Chen, C., He, J., *et al.* (2012). Reciprocal regulation of Akt and Oct4 promotes the self-renewal and survival of embryonal carcinoma cells. Mol Cell *48*, 627-640.

Liu, Q., Ding, C., Liu, W., Song, L., Liu, M., Qi, L., Fu, T., Malovannaya, A., Wang, Y., Qin, J., *et al.* (2013). In-depth proteomic characterization of endogenous nuclear receptors in mouse liver. Mol Cell Proteomics *12*, 473-484.

Uncropped Immunoblots

Figure 1 -- 1B, 1D and 1E
Figure 2 -- 2B, 2C, 2F, 2G, 2H and 2I
Figure 3 -- 3A, 3B, 3C, 3E, 3G and 3H
Figure S12 -- S12B

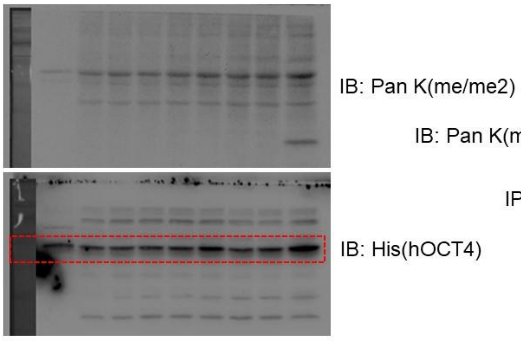

Fig. 1B

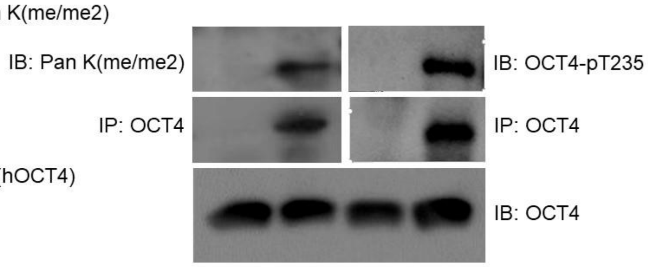

Fig. 1D + 1E

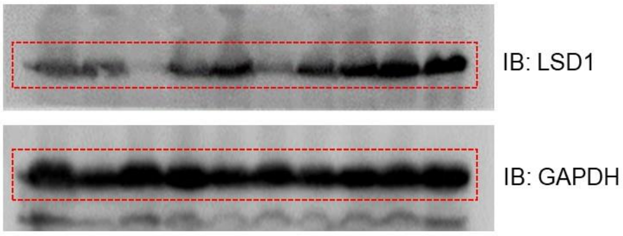

Fig. 2B

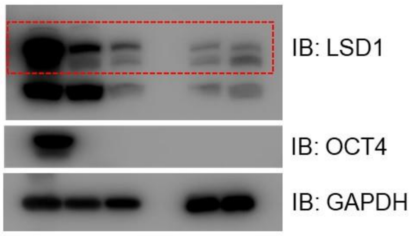

Fig. 2C

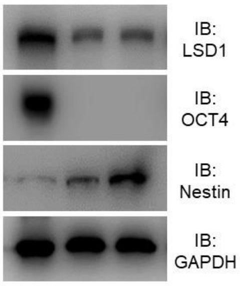

Fig. 2F

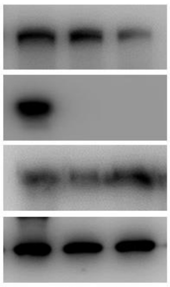

Fig. 2G

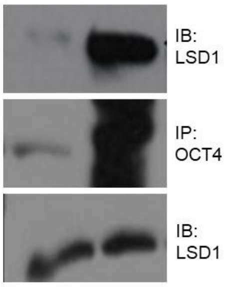

Fig. 2H

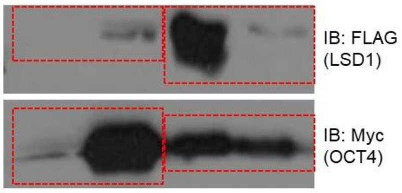

Fig. 2I

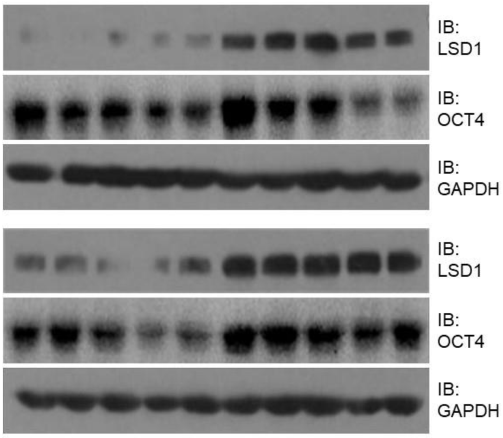

Fig. 3A

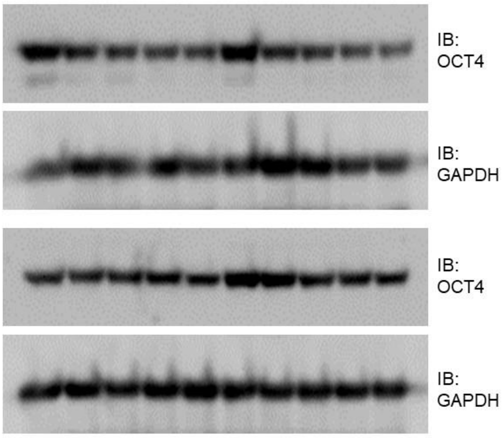

Fig. 3B

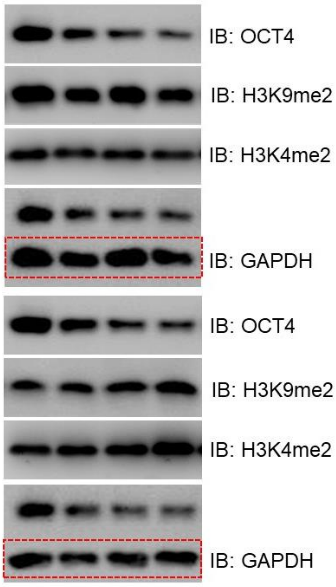

Fig. 3C

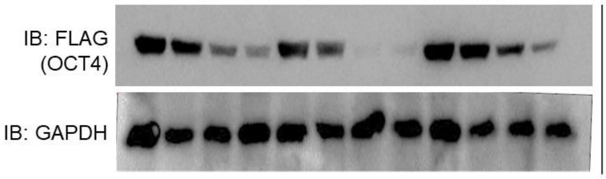

Fig. 3E

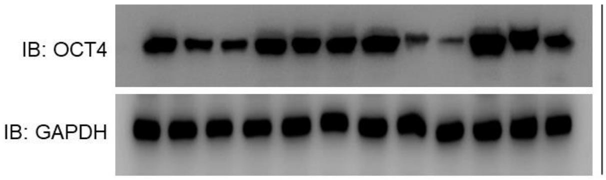

Fig. 3G

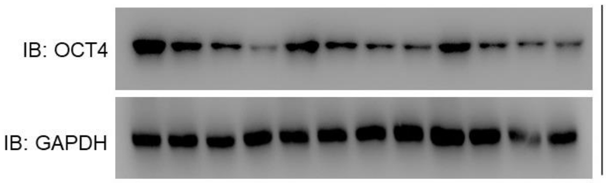

Fig. 3H

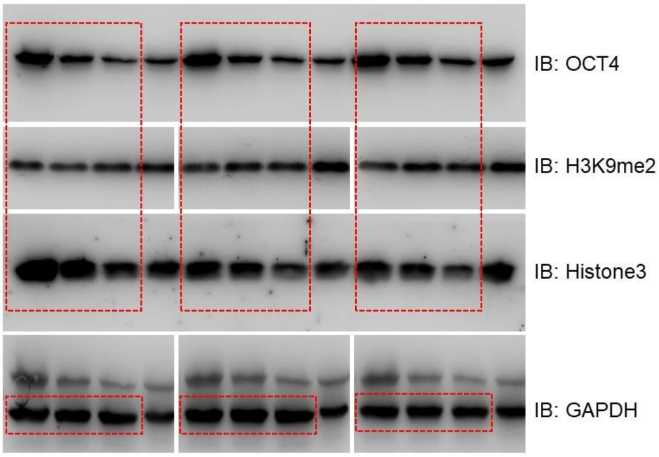

Fig. S12B
